# Integrating Whole-Genome Sequencing Data Into Quantitative Risk Assessment of Foodborne Antimicrobial Resistance: A Review of Opportunities and Challenges

**DOI:** 10.3389/fmicb.2019.01107

**Published:** 2019-05-21

**Authors:** Lucie Collineau, Patrick Boerlin, Carolee A. Carson, Brennan Chapman, Aamir Fazil, Benjamin Hetman, Scott A. McEwen, E. Jane Parmley, Richard J. Reid-Smith, Eduardo N. Taboada, Ben A. Smith

**Affiliations:** ^1^Public Health Risk Sciences Division, National Microbiology Laboratory, Public Health Agency of Canada, Guelph, ON, Canada; ^2^Department of Pathobiology, Ontario Veterinary College, University of Guelph, Guelph, ON, Canada; ^3^Centre for Foodborne, Environmental and Zoonotic Infectious Diseases, Public Health Agency of Canada, Guelph, ON, Canada; ^4^Department of Population Medicine, Ontario Veterinary College, University of Guelph, Guelph, ON, Canada; ^5^National Microbiology Laboratory, Public Health Agency of Canada, Winnipeg, MB, Canada

**Keywords:** next-generation sequencing, risk analysis, genomic epidemiology, food safety, public health, antimicrobial resistance

## Abstract

Whole-genome sequencing (WGS) will soon replace traditional phenotypic methods for routine testing of foodborne antimicrobial resistance (AMR). WGS is expected to improve AMR surveillance by providing a greater understanding of the transmission of resistant bacteria and AMR genes throughout the food chain, and therefore support risk assessment activities. At this stage, it is unclear how WGS data can be integrated into quantitative microbial risk assessment (QMRA) models and whether their integration will impact final risk estimates or the assessment of risk mitigation measures. This review explores opportunities and challenges of integrating WGS data into QMRA models that follow the Codex Alimentarius Guidelines for Risk Analysis of Foodborne AMR. We describe how WGS offers an opportunity to enhance the next-generation of foodborne AMR QMRA modeling. Instead of considering all hazard strains as equally likely to cause disease, WGS data can improve hazard identification by focusing on those strains of highest public health relevance. WGS results can be used to stratify hazards into strains with similar genetic profiles that are expected to behave similarly, e.g., in terms of growth, survival, virulence or response to antimicrobial treatment. The QMRA input distributions can be tailored to each strain accordingly, making it possible to capture the variability in the strains of interest while decreasing the uncertainty in the model. WGS also allows for a more meaningful approach to explore genetic similarity among bacterial populations found at successive stages of the food chain, improving the estimation of the probability and magnitude of exposure to AMR hazards at point of consumption. WGS therefore has the potential to substantially improve the utility of foodborne AMR QMRA models. However, some degree of uncertainty remains in relation to the thresholds of genetic similarity to be used, as well as the degree of correlation between genotypic and phenotypic profiles. The latter could be improved using a functional approach based on prediction of microbial behavior from a combination of ‘omics’ techniques (e.g., transcriptomics, proteomics and metabolomics). We strongly recommend that methodologies to incorporate WGS data in risk assessment be included in any future revision of the Codex Alimentarius Guidelines for Risk Analysis of Foodborne AMR.

## Introduction

Antimicrobial resistance (AMR) represents a major threat to public health, with an estimated 700,000 deaths attributable to AMR every year in the world, and a projected 10 million deaths per year by 2050 in the absence of additional control measures (O’Neill, 2014). AMR is believed to cost approximately 55 billion USD each year in the United States, and some argue this value is underestimated (Smith and Coast, 2013). AMR has been identified as a key priority of the United Nations, as reaffirmed at the General Assembly held in September 2016 where international organizations including the World Health Organization, the Food and Agriculture Organization of the United Nations, and the World Organisation for Animal Health committed to fight AMR together and further collaborate on the implementation of the Global Action Plan on AMR (World Health Organization [WHO], 2015a).

Humans are exposed to antimicrobial-resistant bacteria via food consumption, as well as through animal contact, the environment (including water) and person-to-person contact (Holmes et al., 2016). The presence of antimicrobial-resistant bacteria in food can be due to the use of antimicrobials during agricultural production, to the survival of an antimicrobial-resistant bacteria strain in the food chain (despite little or no antimicrobial use), to the addition of technological bacteria (e.g., starter cultures, probiotics) containing AMR genes (Christoph et al., 2018) or to cross-contamination with antimicrobial-resistant bacteria during food processing and handling (Verraes et al., 2013). The European Food Safety Authority (EFSA) suggests that AMR in food can be addressed either as a direct or an indirect hazard (European Food Safety Authority [EFSA], 2008). A direct hazard refers to the presence of an antimicrobial-resistant pathogenic bacterium in or on food that can colonize or infect people after food ingestion or handling. An indirect hazard is defined as an antimicrobial-resistant bacterium that may transfer resistance genes to a bacterium pathogenic to humans, either directly, or via another commensal bacterium. The AMR gene is the hazard of interest in this case. A bacterium may present both a direct and indirect hazard, e.g., when the AMR gene(s) is carried on a potentially transferable element, such as *Salmonella* carrying plasmid borne extended-spectrum beta-lactamase (ESBL) resistance (European Food Safety Authority [EFSA], 2008). The relative importance of exposure via food versus other routes of transmission is difficult to assess, with major data gaps preventing accurate source attribution of the human burden of AMR (Pires et al., 2018). However, food is likely to be a major route of exposure to antimicrobial-resistant bacteria for common foodborne pathogens, e.g., *Salmonella* or *Campylobacter* (Newell et al., 2010). AMR has been recognized as a foodborne concern since the early 2000s and consequently a number of national food safety authorities, mainly from high-income countries, have implemented routine surveillance of AMR in food (World Health Organization [WHO], 2014). Until recently, AMR surveillance was based on phenotypic methods for antimicrobial susceptibility testing (AST), involving culture on selective or non-selective agar plates, isolation of pure bacterial colonies and subsequent use of disc diffusion, broth dilution, gradient test or other similar methods to determine the inhibition zone diameter or the minimum inhibitory concentration (MIC) for a panel of antimicrobials; the zone diameters or MIC values were subsequently assessed against clinical breakpoints to determine if a bacterial isolate was susceptible or resistant to different antimicrobials (Anjum, 2015).

With the decreasing costs and increasing rapidity and reliability of sequencing technologies, the uptake of next-generation sequencing and especially whole-genome sequencing (WGS) by public health and food safety laboratories has ramped up in recent years, and these new methods are set to replace traditional phenotypic methods for routine surveillance of AMR and other food safety hazards in the near future (Taboada et al., 2017; Oniciuc et al., 2018). In typical WGS protocols, following DNA extraction and shearing into a pool of DNA fragments (i.e., a library) representing the totality of the genome, the library is sequenced in a set of massively parallel sequencing reactions and these are analyzed in a sequencing instrument that can determine the DNA sequence for each fragment in the library (Heather and Chain, 2016). These fragments are later assembled into a draft or complete genome that can be used for further analysis, including gene prediction and annotation (e.g., identification of genes in the genome), comparative genomics (e.g., identification of genome variability, including single nucleotide, allelic variants and differences in gene content) and evolutionary analysis (e.g., generation of trees to depict the evolution of an organism) (Ronholm et al., 2016). A survey conducted by EFSA in 2016 showed that 17 out of 30 European countries already had capacity to perform WGS of foodborne pathogens and 22% of interviewed laboratories had ongoing routine activities involving WGS (European Food Safety Authority [EFSA], 2018). In the United States, the National Antimicrobial Resistance Monitoring System (NARMS) is performing routine WGS analysis of *Salmonella* and *Campylobacter*, in addition to some sequencing of resistant strains of *Escherichia coli* and *Enterococcus* collected from food-producing animals, retail meats and humans (Food and Drug Administration, 2018). Similarly, Canada recently implemented routine use of WGS for surveillance of *Listeria monocytogenes, Salmonella* (since 2017) and *E. coli* (since 2018) collected from agri-food samples and human clinical isolates (Public Health Agency of Canada, 2018).

**FIGURE 1:**
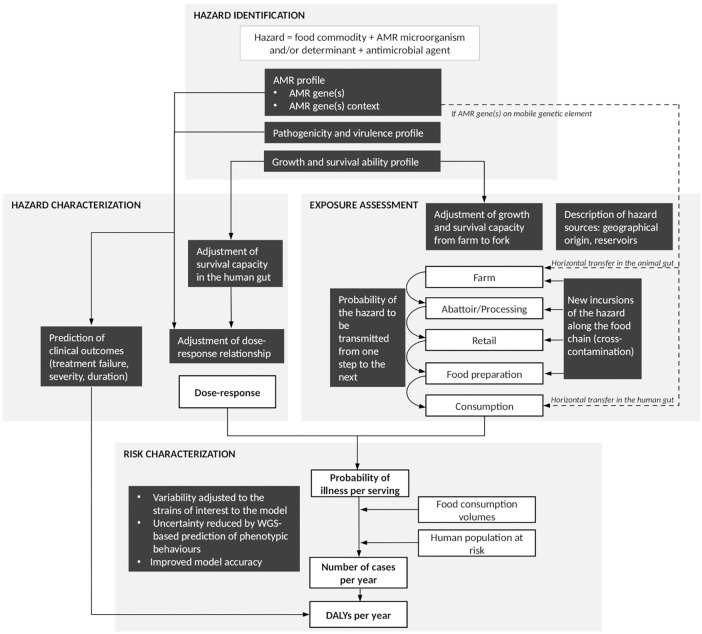
Summary figure of the steps at which whole-genome sequencing (WGS) can contribute to improve quantitative microbial risk assessment (QMRA) of foodborne antimicrobial resistance (AMR). White boxes represent steps of a farm-to-fork risk assessment as conventionally recommended by the Codex Alimentarius Guidelines (Codex Alimentarius, 2011). Black boxes highlight areas where additional pieces of information may be provided by WGS data analysis. Solid arrows: direct connections between elements of the QMRA. Dash arrows: additional connections to be considered in cases where AMR is addressed as an indirect hazard (European Food Safety Authority [EFSA], 2008).

In addition to *in silico* speciation and sub-species level differentiation of isolates (i.e., subtyping), as well as a description of the molecular mechanisms underlying observed resistance phenotypes, the use of WGS is expected to assist AMR surveillance by providing a greater understanding of the transmission of AMR bacteria and genes throughout the food chain, and therefore support risk assessment of foodborne AMR (Food and Drug Administration, 2018; Public Health Agency of Canada, 2018). Quantitative microbial risk assessment (QMRA) models are typically based on the Codex Alimentarius principles for the conduct of microbiological risk assessment and provide a transparent and science-based approach to identify and assess a chain of events that affect the frequency and amount of a microorganism to which humans are exposed through the consumption of food and to describe the magnitude and severity of the adverse health effects from that exposure (Codex Alimentarius, 1999). Basic steps include hazard identification, exposure assessment, hazard characterization and risk characterization (Codex Alimentarius, 1999). In 2011, Codex Alimentarius released specific Guidelines for Risk Analysis of Foodborne AMR (Codex Alimentarius, 2011). These Guidelines, as well as other risk assessment approaches, have been applied to a number of QMRA models of foodborne AMR in the past (McEwen, 2012; Caffrey et al., 2018). All of these models, as well as the Codex Alimentarius Guidelines, were developed prior to the WGS era, and therefore did not include or consider WGS data. Consequently, it is unclear at this stage how WGS data can be integrated into QMRA models of foodborne AMR, and how their integration will impact the QMRA approach and resulting risk estimates compared to using, for example, phenotypic data. Several review papers have addressed the potential use of ‘omics’ data (including genomics, metagenomics, transcriptomics, proteomics and metabolomics) for next generation QMRA (Brul et al., 2012; Bengtsson-Palme, 2017; Den Besten et al., 2018; Haddad et al., 2018; Rantsiou et al., 2018). They included a diversity of genomic techniques and microbial hazards; however, none have explicitly focussed on the use of WGS for QMRA of foodborne AMR. In this literature review, we explore opportunities and challenges of integrating WGS data into QMRA models of foodborne AMR, following the framework proposed in the Codex Alimentarius Guidelines (Codex Alimentarius, 2011). Throughout the text, readers are invited to refer to Figure 1 that summarizes the key aspects presented in the different sections of this review, as well as the connections between sections.

## Hazard Identification

The purpose of hazard identification is to describe the antimicrobial-resistant hazard of concern and evaluate and present the evidence indicating that it is, in fact, a potential risk (Codex Alimentarius, 2011). At this step, risk assessors review literature and information from surveillance programs related to the hazard of interest, typically an antimicrobial-resistant microorganism and/or determinants (i.e., AMR genes) and antimicrobial agents to which resistance is expressed (AMR profile) in a given food commodity (Codex Alimentarius, 2011).

### WGS and AMR Profile

#### WGS and AMR Genes

The use of WGS can refine the description of the AMR profile of a hazard as traditionally provided by phenotypic AST. Bioinformaticians have developed multiple tools to detect the presence of AMR genes in an isolate by comparing its sequence against known genes cataloged in a reference database of known AMR determinants, such as ResFinder or the Comprehensive Antibiotic Resistance Database (CARD). This is typically done using homology-based algorithms such as BLAST (Basic Local Alignment Search Tool) (Anjum, 2015; McArthur and Tsang, 2017). Some prediction tools such as ABRicate, ResFinder or the Search Engine for Antimicrobial Resistance (SEAR) focus exclusively on the detection of acquired AMR genes [i.e., genes acquired from other bacteria via horizontal gene transfer or through the acquisition of mobile genetic elements (MGEs)], while others such as the Antibiotic Resistance Gene-ANNOTation (ARG-ANNOT), Antimicrobial Resistance Identification By Assembly (ARIBA) or Resistance Gene Identifier (RGI) also include resistance that occurred by spontaneous mutation of the genes that encode antimicrobial targets or drug transport systems (Hunt et al., 2017; McArthur and Tsang, 2017). Provided the AMR reference gene database is comprehensive and up-to-date, WGS can provide an exhaustive list of AMR genes present in a given hazard, with no restriction on the number of antimicrobial classes or agents tested simultaneously (this number is typically restricted with phenotypic AST techniques). Based on this detailed molecular profile, the expected AMR phenotype, including the potential for occurrence of multidrug resistance, can be predicted. Some AMR reference gene databases such as CARD provide a list of bacterial species where the AMR gene has been previously found (Jia et al., 2016). Should the AMR gene(s) be selected as the hazard of interest, this information can help risk assessors to define the scope of bacterial species (including pathogenic or commensal bacteria) to be considered as part of the QMRA.

#### WGS and AMR Genes Context

In general, AMR genes can be located either on bacterial chromosomes or on MGEs, allowing AMR gene transmission via clonal spread or horizontal transfer, respectively. MGEs include factors which enable AMR genes to move within or between DNA molecules within the same cell (i.e., insertion sequences, transposons and gene cassettes/integrons), as well as factors which enable AMR genes to be transferred between bacterial cells (i.e., plasmids and integrative conjugative elements) (Partridge et al., 2018). Plasmids appear to substantially contribute to the spread of AMR via food and food-producing animals, particularly among Gram-negative bacteria (Madec and Haenni, 2018). The determination of plasmid sequences has remained a challenge due to the presence of repeated (e.g., insertion sequences) and redundant sequences (e.g., when multiple plasmids are present) which complicate the assembly procedure, especially when using short-read sequencing techniques. The increasing use of long read sequencing technologies has helped to accurately generate plasmid structures, although the costs of these technologies limit their uptake (Orlek et al., 2017). New tools, however, such as the recently developed Recycler and MOB-suite, make it possible to reconstruct and type plasmids with high sensitivity and specificity, although their level of accuracy is still lower than that provided by long read sequencing technologies (Rozov et al., 2017; Robertson and Nash, 2018). The latter tool can also predict the transferability of plasmids (Robertson and Nash, 2018). These developments have facilitated confirmation of whether AMR genes are located on plasmids or integrated into the chromosome (i.e., the AMR genes context), which is a critical piece of information for foodborne AMR risk assessment. If WGS analysis reveals that two or more AMR genes are located on the same genetic element, then co-selection for AMR (i.e., the selection of multiple AMR genes when only one of these genes is selected, for example via antimicrobial use) should be considered in the QMRA model (Wales and Davies, 2015).

#### WGS and AMR as a Direct or Indirect Hazard

As part of the hazard identification, one of the key challenges faced by risk assessors is to decide whether the hazard of interest should be the antimicrobial-resistant microorganism or the AMR gene, or in other words, whether AMR should be considered as a direct or indirect hazard (European Food Safety Authority [EFSA], 2008). WGS can provide information to support this decision. As an example, using WGS of a collection of cephalosporin-resistant *E. coli* from humans, chicken meat, pigs and poultry farms, De Been et al. (2014) demonstrated that clonally unrelated human and poultry isolates carried cephalosporin-resistant genes encoded on genetically identical plasmids (De Been et al., 2014). However, they failed to demonstrate recent clonal transmission of cephalosporin-resistant *E. coli* strains from poultry to humans, as had been suggested based on traditional, low-resolution typing methods (De Been et al., 2014). Clearly, in this case, the cephalosporin resistance gene should be considered as an indirect hazard. Conversely, if clonal transmission is suspected to be the main route of foodborne AMR transmission (after consideration that clonality between isolates collected from animals and humans could still be caused by other routes of transmission besides food consumption), the direct hazard approach may be preferred. Approaches based on AMR foodborne hazards as direct and indirect are both relevant and possible, and the final decision will ultimately depend on the combination of food commodity/AMR hazard/antimicrobial agent and the risk assessment question to be addressed (Pires et al., 2018). A combination of both approaches could also be envisaged.

### WGS and *in silico* Subtyping

Whole-genome sequencing can be used for subtyping of foodborne hazards, and current *in silico* techniques make it possible to do so at a higher speed, lower cost, higher resolution and better robustness when compared to conventional subtyping methods. For example, SISTR [Salmonella In Silico Typing Resource (Yoshida et al., 2016)], SeqSero (Zhang et al., 2015), and EnteroBase (Alikhan et al., 2018) can rapidly subtype draft *Salmonella* genome sequences. Similar tools have been developed for subtyping of other zoonotic hazards such as *E. coli* (e.g., SerotypeFinder, Joensen et al., 2014) or methicillin-resistant *Staphylococcus aureus* (e.g., spaTyper, Bartels et al., 2014). Independent of the AMR profile, knowledge of the subtype of a foodborne hazard is critical for QMRA purposes, as it allows linkages of new data to the huge amount of knowledge already available on the basis of the subtype. Different subtypes (e.g., serotype or sequence type) often have different behaviors or properties that may affect the final QMRA risk estimates, such as different levels of virulence and variability in associated health outcomes (Jones et al., 2008) or different abilities to survive and grow on meat (Oscar, 2009). *In silico* subtyping is also required to assess the degree of relatedness between isolates and understand structures of bacterial populations found at different stages of the food chain; this aspect is discussed further in the exposure assessment section of this review.

### WGS and Pathogenicity or Virulence Profile

Whole-genome sequencing can be used to refine the description of the profile of pathogenicity (i.e., ability to cause disease) or virulence (i.e., severity of that disease) of a given hazard. Prediction tools can be used to detect the presence of known pathogenicity or virulence genes, such as those determining the capacity of attachment, adhesion, invasion or replication of an isolate. As an example, the Center for Genomic Epidemiology (CGE) has developed web-based tools to identify acquired virulence genes among several bacterial species (VirulenceFinder, Joensen et al., 2014), as well as pathogenicity islands among *Salmonella* spp. (SPIFinder, Roer et al., 2016). However, genes providing higher pathogenicity or virulence are not always known *a priori*. In this case, Genome Wide Association Studies (GWAS), also known as Whole Genome Association Studies (WGAS), make it possible to identify genetic markers or genetic risk factors associated with increased pathogenicity or virulence. Briefly, GWAS identify molecular markers such as genes, k-mers, insertions/deletions or single base-pair changes (also called single nucleotide polymorphisms or SNPs) in the DNA sequence of an organism that are significantly associated with a phenotypic trait of interest (Lees and Bentley, 2016; Aun et al., 2018). GWAS present a number of challenges, not only in terms of study design (e.g., identifying well-defined phenotypes, obtaining representative samples and defining optimum sample size), but also in terms of data analysis. It is beyond the scope of this review to address the strengths and caveats of GWAS, but readers are encouraged to consult existing literature for more details (see for example Earle et al., 2016; Falush, 2016).

As an example, Buchanan et al. (2017) conducted a GWAS using 166 *Campylobacter jejuni* isolates representative of the most prevalent subtypes observed in various surveillance projects in Canada and identified 25 genes as putative diagnostic markers for clinically related *C. jejuni* subtypes; these could form a basis for rapidly screening strains that pose an increased risk to public health (Buchanan et al., 2017). Using a similar GWAS approach, Pielaat et al. (2015) identified 17 SNPs significantly associated with increased virulence (using *in vitro* adherence to epithelial cells as a proxy) among 38 Shiga toxin-producing *E. coli* (STEC) O157 isolates of human and animal origin (Pielaat et al., 2015). GWAS have also been applied to the identification of protein families significantly associated with bacterial pathogenicity. Cosentino et al. (2013) used a set of 513 organisms tagged as human non-pathogens and 372 tagged as human pathogens of any bacterial species (i.e., all available complete bacterial genomes from the NCBI-National Center for Biotechnology Information- Genome Project in 2010) to develop a model and a web-based tool that can now be used to predict the pathogenicity of novel species or subtypes toward human hosts (PathogenFinder, Cosentino et al., 2013).

### WGS and Microorganism Growth or Survival Ability

Similarly, WGS can be used to better characterize the ability of a microorganism to grow or survive within a host or in a given environment along the food chain, when challenged with various stress conditions (e.g., cold, heat, acidity, high osmolality, desiccation or use of detergents and disinfectants). Using methods akin to the prediction of AMR and pathogenicity or virulence genes, bioinformatics pipelines can be used to identify the presence of genes known to provide higher ability to grow or survive under stress conditions. As an example, Mandal and Kwon (2017) recently identified 61 genes associated with *Salmonella enterica* serovar Typhimurium survival against desiccation stress; phenotypic evaluation confirmed that three out of 12 single gene knockout mutants had significantly reduced survival as compared to the wild type during desiccation (Mandal and Kwon, 2017). In the absence of known genes encoding for growth or survival ability, GWAS can be used to identify genetic markers for increased growth or survival ability. For example, GWAS studies have been successfully used to identify genetic markers of the ability of *C. jejuni* to form biofilms (Yahara et al., 2017), the ability of multiple *S. enterica* serovars to survive *in vivo* in cattle (Vohra et al., 2018) or the ability of *L. monocytogenes* to grow at low temperatures (Hingston et al., 2017; Fritsch et al., 2018a).

## Exposure Assessment

The objective of the exposure assessment step of a foodborne AMR QMRA model is to arrive at an estimate of the probability and magnitude of exposure to an antimicrobial-resistant microorganism or determinant via consumption of a given food commodity (Codex Alimentarius, 2011). It involves describing the hazard sources and exposure pathways, as well as the risk factors influencing the frequency and concentration of the antimicrobial-resistant microorganism or determinant along the farm-to-fork continuum (Codex Alimentarius, 2011). These tasks can be significantly improved by taking advantage of the high discriminatory power of WGS (i.e., its ability to distinguish between two isolates of the same species and thereby suggest or refute an epidemiological relationship between them) that exceeds conventional phenotypic typing methods (Tassios and Moran-Gilad, 2018). The degree of relatedness between isolates can be assessed using comparative genomics approaches, e.g., SNP-based (where SNPs of aligned genomes are compared) and gene-by-gene approaches (where alleles of 10^2^ to 10^3^ genes are compared between genomes). The latter is an expansion of the traditional multilocus sequence typing (MLST) approach (typically based on allele comparison of seven house-keeping genes) and includes whole-, core- and accessory-genome MLST (wgMLST, cgMLST and agMLST) (Maiden et al., 2013). The strengths and weaknesses of SNP-based vs. gene-by-gene approaches have been discussed elsewhere (Schürch et al., 2018), and it is unknown at this stage which approach should be preferred for QMRA purposes. The optimal approach is likely to depend on the hazard being studied (gene-by-gene approaches may be superior for highly recombinogenic organisms while SNP-based approaches may have more discriminatory power to study highly clonal organisms), as well as other practical considerations (e.g., ability to scale up or standardize analyses for global sharing). Previous applications of SNP-based, wgMLST and cgMLST to the same dataset of foodborne microbial isolates, including *S. enterica* serovar Enteritidis (Pearce et al., 2018), *L. monocytogenes* (Henri et al., 2017) and quinolone-resistant and susceptible *C. jejuni* (Leekitcharoenphon et al., 2018) showed high level of congruence. Another issue relates to the fact that there is currently no consensus on thresholds of relatedness to be used, i.e., the number of SNP or allele differences for two isolates to be considered as significantly different (i.e., belonging to different clusters or lineages). These need to be established on an organism-by-organism and case-by-case basis (Schürch et al., 2018). It requires a good understanding of the underlying population structure and diversity, as well as accurate epidemiological data to be able to link isolates around common sources or timeframes (European Food Safety Authority [EFSA], 2013; Sanaa et al., 2019). As two bacterial populations may share a similar genetic profile due to evolutionary pressures that may not be epidemiologically relevant in the desired context, supporting WGS analyses with strong epidemiological data will remain critical.

### WGS and Sources of Foodborne AMR

The high discriminatory power offered by WGS analysis has the potential to improve source attribution of foodborne AMR, i.e., the attribution of cases of foodborne disease to putative sources of infection (Fegan and Jenson, 2018; Pires et al., 2018), including countries and regions of origin. Using WGS of 502 *C. jejuni* isolates from poultry in 12 European countries and a set of 536 previously published *C. jejuni* genomes retrieved from the European Nucleotide Archive, Leekitcharoenphon et al. (2018) were able to examine the origin of fluoroquinolone resistance among *C. jejuni*. In other words, they explored whether the emergence of fluoroquinolone-resistant *C. jejuni* was related to the transmission among countries or to the selection through fluoroquinolone use in individual countries (Leekitcharoenphon et al., 2018). Gene-by-gene analysis of isolate relatedness showed that poultry *C. jejuni* populations were clustered within four groups of countries of origin, but no significant association was observed with poultry trade patterns or antimicrobial use in livestock (Leekitcharoenphon et al., 2018). Similarly, a SNP-based phylogeny of 90 multidrug-resistant *S. enterica* genomes of human and dairy cattle origin, collected in Washington (WA) and New York (NY) in the United States, highlighted several geographic location-specific clones (e.g., a WA specific *S. enterica* serovar Dublin clade, which likely emerged recently from this particular location), as well as broadly distributed clonal groups with similar AMR profiles that likely emerged a long time ago and successfully disseminated to wider populations (Carroll et al., 2017).

Whole-genome sequencing can moreover inform on host species and reservoirs of foodborne AMR. Using WGS of 113 cephalosporin-susceptible and resistant *S. enterica* serovar Heidelberg isolates from human and poultry origin collected under the Canadian Integrated Program for Antimicrobial Resistance Surveillance (CIPARS), Edirmanasinghe et al. (2017) showed that most human isolates clustered with retail chicken isolates, while the observed degree of relatedness with retail turkey isolates was minor, suggesting a chicken-origin of human *S.* Heidelberg infections in Canada (Edirmanasinghe et al., 2017). Such analysis would not have been possible with conventional typing approaches [e.g., pulsed-field gel electrophoresis (PFGE) or MLST] that have insufficient discriminatory power to distinguish microorganisms with low genetic diversity such as *S.* Heidelberg. Similarly, Carroll et al. (2017) performed a comparative genomics analysis of livestock- and human-associated *Salmonella* from WA and NY and showed overlap between the resistomes of bovine and human-associated *Salmonella* isolates on numerous occasions, particularly for *S. enterica* serovar Newport, suggesting a bovine origin of *S.* Newport human infections (Carroll et al., 2017). In comparison with traditional typing tools such as PFGE or MLST, phylogenetic analyses based on WGS data make it possible not only to demonstrate shared patterns of AMR in pathogens from animals, meat and humans with high confidence, but also to infer the direction of transmission (Muloi et al., 2018). While traditional typing tools could already identify overlapping subtypes, the significance of such overlaps was directly influenced by the subtype frequency. WGS allows for splitting the bacterial population into more subtypes (including more rare subtypes), so any overlaps observed are unlikely to be spurious.

The major sources identified can be used as starting points of QMRA models (traditionally focusing on a single source only) or comparative exposure or risk assessments (that consider exposure from multiple primary sources or multiple pathways from a single animal source, see for example Pintar et al., 2017; Chapman et al., 2018). The use of WGS for source attribution is still in its infancy, and to our knowledge has not yet been applied to source attribution of antimicrobial-resistant hazards. Preliminary attempts to refine source attribution of human cases of campylobacteriosis using wgMLST demonstrated the challenge of identifying host-segregating genetic markers (Dearlove et al., 2016; Thépault et al., 2017), although these may be present in the accessory genome of *Campylobacter* (Sheppard et al., 2013). These attempts may have partly been hampered by the complexity of *Campylobacter* transmission in the food chain (e.g., interactions between cattle and chicken reservoirs). WGS was successfully used for source attribution of *L. monocytogenes* in the European Union (Nielsen et al., 2017). Further work is needed to explore other approaches and applications to other pathogens of public health interest. The uptake of WGS for source attribution also faces a non-scientific challenge; increased resolution of WGS data may inadvertently identify individual producers as sources of foodborne AMR. While such an attribution may be appropriate (and indeed beneficial) in investigations of foodborne outbreaks, a lack of corresponding epidemiological evidence to support or refute such a connection at the scale of source attribution may inappropriately place blame on a non-epidemiologically linked producer. Concern regarding liability may limit the number of samples submitted in voluntary surveillance programs, decreasing their overall effectiveness.

### WGS and Exposure Pathways of Foodborne AMR

Once the sources of foodborne AMR have been identified, WGS can be used to refine the description of the exposure pathways, i.e., the pathways through which a hazard is transmitted from source (e.g., farm) to point-of-exposure (e.g., consumption). This can be done using comparative genomics of isolates collected at successive points or stages of the farm-to-fork continuum (Figure 1). Such an approach has already been proposed for outbreak investigations. For example, the United States Food and Drug Administration (FDA)’s Center for Food Safety and Applied Nutrition (CFSAN) outbreak investigation framework considers: (i) the genetic distances between isolates identified on the basis of the number of SNPs; these are interpreted by taking into account previous knowledge on the diversity and evolutionary forces of the pathogen population of interest; (ii) the uncertainty around genetic distances which is assessed using bootstrapping; (iii) the topology of the SNP-based phylogenetic tree, with monophyletic trees (i.e., those grouping isolates of interest to the exclusion of all other isolates) supporting the hypothesis of a common source and (iv) epidemiological and traceback data (Pightling et al., 2018). We argue that a similar framework could be developed for QMRA of foodborne AMR, with the objective of describing the probability of a hazard being transmitted from one step of the food chain to the next. A few adjustments to the framework proposed by FDA CFSAN would be required, as described below.

First, sampling performed at the different stages of the food chain has to be large enough and representative of the bacterial populations present at these stages. FDA CFSAN WGS analyses rely on the GenomeTrakr database, an open-source collection of genomic and geographic data about foodborne pathogens submitted by public health and university laboratories across the United States (Allard et al., 2016). Although the largest and most complete of its kind, FDA CFSAN recognizes that this database is likely biased toward food isolates and environmental isolates from facilities that yield positive results (just like any outbreak-based database) (Pightling et al., 2018). Representative samples can, however, be obtained from epidemiological surveillance programs. For example, CIPARS is designed to provide a representative sample of antimicrobial-resistant *Campylobacter, E. coli* and *Salmonella* populations circulating on-farm, at abattoir and at retail for major livestock species or their meat products (Deckert et al., 2015). However, obtaining a representative sample of antimicrobial-resistant and susceptible bacterial populations found in humans is a challenge, owing to underreporting and under-diagnosis, as well as bias toward more severe clinical cases that have a higher chance of being investigated and reported to health authorities (Haagsma et al., 2013).

Second, a quantitative outcome describing the probability of a hazard to be transmitted from one step of the food chain to the next is needed. Pightling et al. (2018) use qualitative outcomes to assess whether each of their four criteria supports, is neutral or does not support a match between two or more genomes obtained by WGS (Pightling et al., 2018); this would not be sufficient for QMRA purposes. A quantitative surrogate for the probability of a hazard to be transmitted from one step to the next could be the proportion of genomes found at step *(n+1)* clustering with those found at step *n*. The sensitivity of this probability estimate as a function of the clustering threshold could also be explored. The uncertainty around the probability estimate could be assessed using bootstrapping as proposed by Pightling et al. (2018), and included into a QMRA using stochastic modeling (e.g., Monte Carlo simulation).

In addition to the proportion of hazards being retained at each step, new incursions of the hazard of interest along the food chain are also relevant to QMRA modeling (Figure 1). These typically arise from cross-contamination between or within farms (e.g., because of poor biosecurity practices), during carcass processing (e.g., contamination from the abattoir environment or personnel) or during handling and preparation of the final food product (e.g., poor kitchen hygiene practices) (Carrasco et al., 2012). For example, comparative genomics of bacterial populations found in the abattoir environment with those found on carcasses in the abattoir could be used to inform the probability of cross-contamination occurring from the abattoir environment to the carcasses; this quantity is typically difficult to inform using phenotypic data (Nauta et al., 2005). In light of the large bacterial diversity present in such an environment, a large sample size would, however, be critical for this type of analysis. A proof of concept on practical use of WGS to define the entry routes and spread patterns of *L. monocytogenes* in a meat establishment has already been described (Nastasijevic et al., 2017). Massive cross-contamination of broiler carcasses with ESBL producing-*Klebsiella pneumoniae* and -*E. coli* during scalding and defeathering was also demonstrated using WGS phylogenetic analyses of isolates found on slaughterhouse machinery and carcasses (Projahn et al., 2019).

Finally, the epidemiological data used to support the interpretation of WGS comparative genomics findings should be based on those available from surveillance programs (e.g., year, region, food commodity). Just like any other molecular comparative analyses, direct interpretation of the observed relationships may be hampered by the complexity of the food chain, with food products being increasingly produced, processed, transformed and consumed in different regions or countries and within different timeframes (as influenced by the product shelf life) (Aung and Chang, 2014).

As a simple, deterministic example, if WGS analyses of surveillance data revealed that only half of direct hazards in retail beef are genetically related to isolates collected in on-farm cattle, then the probability of the hazard to be transmitted from the farm to retail product would be 50%. This would have profound implications of the evaluation of interventions in the exposure assessment, as interventions modeled on-farm would only have an impact on 50% of the population of the hazard that humans are exposed to in retail beef, in this hypothetical example (assuming that the other 50% of the hazard population sampled in retail beef have a different origin). Therefore, in a purely linear model, any intervention that reduces prevalence of the hazard by 90% on-farm would only reduce overall exposure to humans from beef consumption by 45%.

### Exposure Assessment and Horizontal Transfer of AMR

In those cases where risk assessors decide to focus on the AMR gene(s) as the hazard of interest, and if AMR profiling via WGS shows that the AMR gene(s) are located on MGEs (e.g., plasmids), then horizontal transfer of AMR genes should preferably be included in the exposure assessment step of a QMRA. This will contribute to providing a comprehensive depiction of the exposure pathways (Figure 1). Horizontal transfer of AMR genes between bacteria can occur via three main mechanisms, namely transformation (uptake of naked DNA), transduction (transfer by bacteriophages) and conjugation (transfer by plasmids and other conjugative elements) (Boerlin and Reid-Smith, 2008). Conjugation, in particular, seems to play an important role in the transmission and spread of foodborne AMR of public health importance (Madec and Haenni, 2018). The majority of plasmid conjugation events likely occur within the gastrointestinal tract of live animals or humans, where bacteria are present in high concentrations and in close proximity to each other, all within optimal survival conditions (although some plasmid groups do not transfer at body temperature). Plasmid conjugation in biofilms (e.g., transfer of plasmids encoding extended-spectrum cephalosporin resistance between *E. coli* and from *E. coli* to environmental bacteria in the food-processing chain) has also been described (Mo et al., 2017). Plasmid conjugation during cooking is likely minimal; no plasmid conjugation was observed from antimicrobial-resistant *E. coli* heated to 60°C for 10 or more minutes (Le Devendec et al., 2018). Following food ingestion, horizontal transfer of MGEs from foodborne antimicrobial-resistant bacteria to commensal or pathogenic bacteria in the human gut can occur, facilitated by exposure to antimicrobials (Broaders et al., 2013; Huddleston, 2014). For example, in an *in vivo* study, an *Enterococcus faecium* isolate from chicken origin transferred a gene coding for vancomycin-resistance (*vanA*) to a vancomycin-susceptible *E. faecium* of human origin in the intestines of three out of six human volunteers (Lester et al., 2006).

Mathematical models describing the dynamics of plasmid-mediated AMR within an animal gut (e.g., ceftiofur-resistant *E. coli* in the large intestine of cattle) that include horizontal transfer of plasmids between bacteria in the presence or absence of antimicrobial treatment have been developed (Volkova et al., 2013), but their integration with between-animal transmission models is needed before these can be included into QMRA models. The lack of quantitative data makes these approaches difficult to generalize to other food commodity/AMR hazard/antimicrobial agent combinations. More generally, for horizontal transfer of AMR genes to be modeled in the exposure assessment step of a QMRA, quantitative data are needed about the direction and frequency of AMR gene transfer occurrence under various physiological conditions, its dependence on the concentrations of the donor and recipient bacteria, as well as the influence of concomitant antimicrobial treatment. These data are currently not provided by WGS analyses, but can be generated through *in vivo* or *in vitro* experiments of horizontal transfer of AMR genes (Poppe et al., 2005; Card et al., 2017; Mo et al., 2017).

Still, WGS can provide useful information to better characterize horizontal transfer of AMR genes. WGS cannot only identify whether AMR genes are located on the chromosome or on MGEs (e.g., plasmids) which is a critical piece of information, but also help to better characterize those plasmids. For example, WGS can be used to identify the incompatibility group or replicon type of a plasmid, which usually correlates with the dynamics and efficiency of horizontal transfer, as well as the plasmid host-spectrum (Partridge et al., 2018). WGS can also explore whether a plasmid has the molecular machinery for conjugation to occur and therefore predict the mobility profile of a plasmid. In other words, WGS can tell us how likely plasmid-mediated AMR genes are transferred horizontally. Bioinformatics tools such as MOB-suite facilitate characterization of plasmid transfer by offering a set of tools to perform plasmid reconstruction, typing and mobility assessment (Robertson and Nash, 2018).

## Hazard Characterization

As part of the hazard characterization step of a foodborne AMR QMRA, risk assessors aim to translate levels of exposure to the hazard into a probability of one or more adverse health outcomes in humans (Codex Alimentarius, 2011). Wherever possible, this involves defining a dose–response relationship, i.e., a mathematical relationship between the exposure and probability of adverse outcomes. Adverse outcomes may include an array of health effects or clinical outcomes, such as infection or disease, as well as additional consequences due to exposure to an antimicrobial-resistant pathogen, such as treatment failure, increased severity or duration of disease and death (Codex Alimentarius, 2011).

### Adjustment of the Dose–Response Relationship

For a foodborne hazard to cause an adverse outcome, bacteria need to survive passage through the human gastrointestinal tract, which presents multiple barriers (e.g., saliva, low pH and pepsin in the stomach, commensal bacteria, enzymes and the innate and adaptive immune systems in the intestine) before reaching a suitable site for colonization, attachment and invasion that will eventually lead to infection and subsequently to disease (Rahman et al., 2016, 2018; Wijnands et al., 2017). Dose–response relationships typically capture these steps together within a single mathematical equation. They are largely based on human clinical experiments (Teunis et al., 1996) or foodborne outbreak data (Teunis et al., 2010) which do not enable modelers to distinguish between the different in-host steps. These sources also aggregate data at the species level, with no consideration to differences between strains of a given hazard. However, using an *in vitro* model of the human gastrointestinal tract, Wijnands et al. (2017) showed that within- and between-strain survival of *S.* Heidelberg and *S.* Typhimurium in the gastrointestinal tract and ability to cause infection was highly variable (Wijnands et al., 2017). WGS profiling of the survival ability of a hazard (as described in the hazard identification step) could be used to refine the prediction of the number of cells (i.e., dose) expected to survive passage through the gastrointestinal tract and to contribute to infection (Figure 1).

**FIGURE 2:**
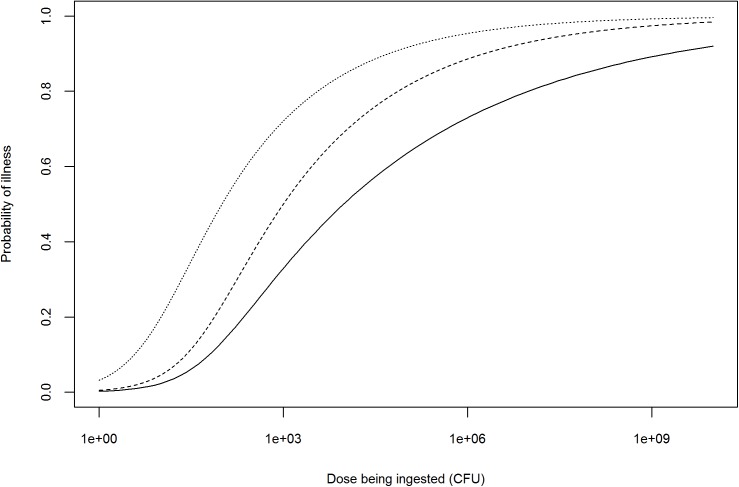
Illustration of possible adjustments to the dose–response relationship of an antimicrobial-resistant *Salmonella* spp. strain based on information provided by WGS profiling. Solid line represents the dose–response curve of a generic *Salmonella* spp. (FAO/WHO, 2002); dashed line: dose–response after theoretical adjustment for high pathogenicity; dotted line: dose–response after theoretical adjustment for high pathogenicity and prior antimicrobial use in human patient.

Once the dose is adjusted, the probability of infection (or disease) could further be refined using WGS profiling of the pathogenicity of the hazard, with highly pathogenic strains requiring lower doses to cause infection or disease (Figure 2). However, the issue of establishing a quantitative relationship between the presence of pathogenicity markers and the probability of infection (or disease), i.e., translating information on hundreds or thousands of genetic markers (e.g., SNPs or differences in gene content) to dozens of biologically relevant effects to a single measure of response (e.g., probability of infection or disease) needs further exploration (Pielaat et al., 2015; Haddad et al., 2018). Probability of infection (or disease) is not linearly related with the number of pathogenicity markers, but also depends on their biological function and levels of expression. Depending on the scope of the QMRA, either a single dose–response relationship (characterizing the survival and pathogenicity profile of the strain of interest) or a combination of several dose–response relationships (based on the distributions of the survival and pathogenicity profiles of the strains of interest) may be used (Chen et al., 2006, 2011; Fritsch et al., 2018b).

Whole-genome sequencing AMR profiling of a hazard can also be used to adjust the dose–response relationship depending on the occurrence of antimicrobial use in humans (i.e., consumers) prior to ingestion of a contaminated product. This refers to the notion of ‘etiologic fraction’ for prior antimicrobial use, defined by the Institute of Medicine as the proportion of human cases that would not occur but for the resistance of the infecting bacterial strain to the antimicrobial(s) being administered to the patient prior to infection (Institute of Medicine, 1989). Human use of antimicrobials prior to ingestion of an antimicrobial-resistant hazard can increase the probability of infection (or illness) by two means: (i) the selection of hazard strains resistant to the antimicrobial being taken prior to infection for an unrelated reason (selective effect) and (ii) the reduction of competing commensal gastrointestinal flora (competitive effect) (Barza and Travers, 2002). As an example, this approach was used by Otto et al. (2014) to assess the excess of human cases of ceftiofur-resistant *S.* Heidelberg in Canada attributable to prior antimicrobial consumption in humans (Otto et al., 2014). However, to our knowledge, no literature suggests that the dose–response relationship of an antimicrobial-resistant hazard would differ from a non-resistant hazard in the absence of antimicrobial treatment (assuming they have similar pathogenicity profiles). The adjustment of the dose–response relationship would therefore only be needed in case of prior antimicrobial use in the human patient, unless WGS reveals an association between AMR and pathogenicity (Figure 2).

While WGS data can provide more accurate estimates of the dose contributing to infection (after survival in the gastrointestinal tract), and better characterize the expected response (based on pathogenicity and AMR profiles), WGS focuses on the characterization of individual isolates and therefore cannot provide any quantitative estimates of the dose being ingested initially (e.g., the dose in a food sample at retail). Other genomic approaches such as metagenomics (i.e., the study of the collective genome of microorganisms from a sample), is likely to help in this regard. For example, bioinformatic analytical pipelines, such as Resfinder, SEAR or AMRplusplus can be used to quantify the abundance of AMR genes in metagenomes of diverse origin, e.g., animal feces or farm effluents (Rowe et al., 2015; Lakin et al., 2017; Munk et al., 2018). However, they only provide relative abundance estimates within samples, whereas absolute estimates are more informative for QMRA modeling purposes. Additionally, the information on host microorganism is lost, so it is unknown whether the genes are carried by the hazard(s) of interest and therefore contribute to risk or not (Cocolin et al., 2018). Alternatively, real-time polymerase chain reaction (qPCR) has been used to quantify the absolute amount of ESBL (bla_TEM_) gene copies present in food, but there are currently no data to convert a number of gene copies into a probability of infection or illness (Singh et al., 2016).

### Prediction of Health Outcomes

Not all cases of foodborne AMR disease are equal, and health outcomes are known to differ substantially among bacterial strains. For example, analysis of epidemiological data from 46,639 *Salmonella* infections between 1996 and 2006 in the United States revealed significant differences among serovars in terms of case-fatality rates, hospitalization rates and ability to cause invasive disease (Jones et al., 2008). Health outcomes also differ between antimicrobial-resistant and susceptible bacteria. A recent systematic review and meta-analysis showed that multidrug-resistant non-typhoidal *Salmonella* infections in high-income countries were associated with excess bloodstream infections and higher hospitalization and mortality rates when compared with pan-susceptible isolates (Parisi et al., 2018). More generally, infections with certain food commodity/AMR hazard/antimicrobial agent combinations have been associated with more severe clinical outcomes, longer duration of infections and treatment failures, when compared to non-resistant hazards (European Food Safety Authority [EFSA], 2008).

Whole-genome sequencing can help to define the array of adverse health effects or clinical outcomes to be expected from infection with the strains of interest in a foodborne AMR QMRA (Haddad et al., 2018). As described earlier (see hazard identification section), WGS virulence profiling can be used to predict the severity of health outcomes to be expected upon infection with a given strain (Figure 1). For example, clinical outcomes associated with *Campylobacte*r human infections include a broad spectrum of symptoms ranging from mild, moderate and severe diarrhea to Guillain-Barré syndrome and even death (World Health Organization [WHO], 2015b). WGS could be used to predict the distribution of these clinical outcomes for a given set of strains under the scope of a QMRA, and therefore refine the distributions typically obtained from epidemiological data that combine data on multiple strains and which have some methodological issues, e.g., underreporting and selection bias (Majowicz et al., 2014).

Similarly, WGS AMR profiling could be used to predict the AMR phenotypic profile of a foodborne hazard upon infection, and therefore predict the range of AMR-associated health outcomes to be expected with or without human antimicrobial treatment (Figure 1). For example, in the case of third-generation cephalosporin-resistant and fluoroquinolone-resistant *E. coli* infections, WGS could help to predict the probability of increased mortality, length of stay in hospital or intensive care unit admission associated with resistant *E. coli* infection when compared to non-resistant *E. coli* (World Health Organization [WHO], 2014). Once the array and distribution of health outcomes are defined, they can be combined into indicators of burden of illness, such as disability adjusted life years (DALYs), that capture not only the number of cases in a given period of time, but also their severity (e.g., disability weight, mortality) and duration (Murray, 1994). These can be used to assess the extra burden associated with AMR foodborne hazards compared with susceptible ones.

## Risk Characterization

Risk characterization integrates the findings from the hazard identification, exposure assessment and hazard characterization steps to estimate the public health risk associated with foodborne AMR (Codex Alimentarius, 2011). Risk measures encompass the severity and likelihood of human infections associated with foodborne AMR, and may be expressed using multiple metrics, such as per-meal risk (e.g., probability of illness per serving), population or annual risk based on consumption (e.g., number of cases per year), as well as burden of illness estimates (e.g., DALYs) (Figure 1) (Codex Alimentarius, 2011). A quantitative description of the variability (i.e., heterogeneity due to quantities that are distributed within the population) and uncertainty (i.e., model-specification error due to lack of knowledge or data) around the risk estimates, as well as an explicit depiction of the strengths and limitations of the QMRA model are warranted at this stage. Sensitivity analysis should also be conducted to identify those parameters having the largest influence on the model outcomes. Important data gaps should be identified and highlighted as future research needs. Additional outputs of risk characterization may include scientific evaluation of possible risk management options (Codex Alimentarius, 2011).

### WGS and Variability in Risk Estimates

As discussed earlier in the hazard identification step, WGS comes with new tools that make it possible to substantially refine the profiling of a hazard of interest in a foodborne AMR QMRA, switching from a taxonomic-based approach (i.e., focusing on specific organisms) to a cluster-based (i.e., focusing on sequence types or population groups) or a strain-based approach (i.e., focusing on particular genes or genetic markers) (Brul et al., 2012; Den Besten et al., 2018). Instead of considering all strains from a particular hazard as equally able to cause disease, WGS provides information on the presence of genes or genetic markers of interest that will allow risk assessors to (i) narrow down the risk assessment by focusing on those strains of highest public health relevance (e.g., those showing highest virulence in humans) and/or (ii) stratify the risk assessment, i.e., grouping hazards into subsets with similar genetic profiles that are expected to behave similarly, e.g., in terms of growth/survival ability along the food chain, pathogenicity/virulence following ingestion and response to antimicrobial treatment. Combinations of genes or genetic markers of interest shall also be considered. For example, AMR and virulence genes have been shown to be carried by the same plasmids found in *S.* Heidelberg of human and animal origin (Han et al., 2012). Expression of risk characterization results should therefore be based on combinations identified in the hazard identification stage, elucidated from simultaneous WGS-based profiling for AMR, growth/survival and pathogenicity/virulence. Using WGS, the entire genetic variability observed in key traits of the strains of interest can therefore be captured without including unnecessary variability (i.e., excluding variability arising from strains not relevant to the QMRA model). The distribution of QMRA model parameters can be adjusted accordingly so that they better fit with the subsets of hazards of interest, improving the overall model accuracy. As an example, Fritsch et al. (2018b) identified genetic subsets of *L. monocytogenes* isolates based on their ability to grow at low temperature, as well as their level of virulence, and were able to refine an existing QMRA model for *L. monocytogenes* in smoked salmon initially based on phenotypic data (Fritsch et al., 2018b).

### WGS and Uncertainty in Risk Estimates

The identification of genes or genetic markers for certain parameters of interest in foodborne AMR QMRA modeling (e.g., AMR, growth/survival and pathogenicity/virulence) via WGS makes it possible to predict the phenotypic behavior of a hazard, and therefore to adjust the model parameters accordingly. For example, predicting the hazard’s ability to grow during processing, transport and storage will provide a more accurate estimate of the number of cells ingested at the point of consumption. Consequently, incorporation of WGS data into QMRA models will help reduce the uncertainty in the final risk estimates. Yet, there is some uncertainty in the degree of correlation between genotypic and phenotypic profiles. The detection of genes or genetic markers does not mean this gene or genetic marker will be functional or expressed. Previous literature has shown very high correlation (i.e., close to 100%) between AMR WGS and phenotypic profiles among *E. coli* (Stoesser et al., 2013; Shelburne et al., 2017), *Staphylococcus aureus* (Gordon et al., 2014), *Campylobacter* spp. (Zhao et al., 2016) and non-typhoidal *S. enterica* (Neuert et al., 2018). WGS even showed high correlation with MIC values in non-typhoidal *Salmonella* (Tyson et al., 2017). However, correlations between AMR WGS and phenotypic profiles still need to be validated on a larger scale and for other zoonotic hazards. Similarly, correlations between WGS and phenotypic profiles in relation to growth/survival ability as well as pathogenicity/virulence have yet to be validated. Unlike AMR, which is easy to test for phenotypically, tests for accurate prediction of pathogenicity or virulence are lacking. The correlation between various parameters thought to impact pathogenicity and virulence and actual outcomes is tenuous for many pathogens. For example, Pielaat et al. (2015) use *in vitro* adherence to epithelial cells as a proxy for virulence in *E. coli* O157:H7, but recognize it is only one aspect of the etiology of *E. coli* O157:H7 human infections among many other aspects, such as the production of Shiga toxins (Pielaat et al., 2015).

Another major source of uncertainty introduced with the use of WGS data for QMRA modeling, and especially for exposure assessment, are the cut-off values used to describe relatedness between isolates. As mentioned earlier, there is currently no consensus on distance thresholds used to define lineages of interest and these need to be empirically determined on an organism-by-organism and case-by-case basis (Schürch et al., 2018). As a general rule, more stringent cut-off values should be used for organisms with lower genetic diversity. For example, Public Health England uses three thresholds of 0-, 5-, and 10-SNP differences for defining an outbreak cluster of isolates under the UK surveillance program for *S.* Enteritidis and *S.* Typhimurium, both being highly clonal pathogens (Mook et al., 2018). These ‘empirical’ thresholds are based on observed SNP differences between isolates known to be associated within outbreaks according to historical epidemiological data, in the absence of any better rule. For QMRA purposes, a range of thresholds capturing the uncertainty around the degree of relatedness between isolates can be used and integrated into a QMRA model using a combination of scenario-based and stochastic approaches. In the future, the use of WGS for outbreak investigation and its increasing use in surveillance of foodborne pathogens will likely improve our knowledge of the structure of bacterial populations, and the collection of isolates from large and representative samples of the global population into open-source databases will provide a scientific basis to define appropriate distance thresholds, i.e., combining good epidemiological concordance (i.e., ability to group epidemiologically related isolates) and discriminatory power (i.e., ability to distinguish non-epidemiologically related isolates) (Van Belkum et al., 2007; Hetman et al., 2017). The uncertainty around the degree of relatedness between isolates, and therefore around risk estimates, will decrease accordingly.

### WGS and Risk Management Options of Foodborne AMR

A number of risk management options can be considered to mitigate the risk associated with the occurrence of AMR in the food chain (Murphy et al., 2018). One of the most promising options is reducing the use of antimicrobials in food-producing animals (Tang et al., 2017). However, quantifying the relatedness between antimicrobial use and the occurrence of AMR using phenotypic data is a difficult task, especially when using surveillance data that are often aggregated at a low resolution level, typically following trends in overall food-animal populations over time, with no details on how antimicrobial use and antimicrobial-resistant bacterial populations are distributed at the farm level. The importance of this relatedness appears to be highly variable between food commodity/AMR hazard/antimicrobial agent combinations and is influenced by co-selection of AMR (i.e., when an antimicrobial selects for resistance to another antimicrobial) (Dorado-García et al., 2016).

As an example, CIPARS reported that changes in the frequency of isolates resistant to ceftiofur (a third-generation cephalosporin antimicrobial) among chicken *S.* Heidelberg isolates collected at retail during 2003–2008 in Québec mirrored the trends in ceftiofur use in hatcheries, with a significant decrease in resistance (from 62 to 7%) following a voluntary withdrawal of ceftiofur use, and an increase in resistance (from 7 to 20%) after reintroduction of ceftiofur use (Dutil et al., 2010). These observations were based on phenotypic data, and the degree of relatedness between ceftiofur-resistant *S.* Heidelberg populations over time was unknown. As *S.* Heidelberg has low genetic diversity, traditional typing methods (e.g., PFGE) are unlikely to provide additional information. Comparison of WGS profiles of *S.* Heidelberg populations over time, however, would enable the assessment of the effect of withdrawing ceftiofur use on the prevalence of ceftiofur-resistant *S.* Heidelberg in broiler chicken meat in Canada to be refined. For example, WGS could help to describe whether a single bacterial population underwent resurgence after reintroduction of ceftiofur use, or whether the original ceftiofur-resistant *S.* Heidelberg population died out and a second population took over after ceftiofur use reintroduction.

## Challenges and Future Perspectives

### Bridging the Gap Between Bioinformaticians and Risk Assessors

The integration of WGS data into QMRA modeling represents a critical step toward the development of next generation QMRA of foodborne AMR. Yet, there are a number of challenges that need to be addressed before the transition can be effective. There is still a lack of knowledge translation between bioinformaticians and risk assessors, with bioinformaticians having a limited understanding of the type of information required for QMRA modeling, and risk assessors having poor comprehension of how WGS data are analyzed, including what type of information can be generated and any limitations on these analyses. Risk assessors and epidemiologists need to be better trained in next generation sequencing techniques and molecular epidemiology (De Lamballerie, 2009; Arts and Weijenberg, 2013). Risk assessors also have to be proactive and should be involved from the early stages of development of WGS databases and bioinformatics tools, so that they can make their needs more explicit and influence the type and format of data being generated, rather than simply acting as ‘end-users’ of WGS data. Input from both bioinformaticians and risk assessors should also be considered while designing surveillance programs and data collection initiatives to ensure these are capturing the data needed to perform the types of analyses described in this review.

Additionally, bioinformaticians should continue to work toward making their WGS analytical tools and pipelines readily available for use by non-bioinformaticians. There are already a number of initiatives in this direction. As an example, the SISTR platform^1^ combines several free tools for rapidly performing simultaneous analyses of draft *Salmonella* genome assemblies, including serovar prediction and sequence-based typing analyses (e.g., cgMLST), as well as metadata driven visualization to examine the phylogenetic, geospatial and temporal distribution of genome-sequenced isolates in comparison with a database comprising over 4,000 publicly available genomes (Yoshida et al., 2016). Similarly, the Center for Genomic Epidemiology offers a number of free tools for WGS genome assembly and rapid analyses within a single online platform, including typing, phenotyping, and phylogenetic tree construction^2^. GenomeGraphR presents WGS and metadata in a user-friendly interface to “query a variety of research questions such as, transmission sources and dynamics, global reach, and persistence of genotypes associated with contamination in the food supply and foodborne illness across time or space” (Sanaa et al., 2019). Enterobase (Alikhan et al., 2018) and PHYLOViZ (Ribeiro-Gonçalves et al., 2016) are two other examples of publicly available platforms facilitating WGS data analysis and visualization by both bioinformatician and non-bioinformatician users. The development of such tools requires an interdisciplinary environment where bioinformaticians can work closely with end-users (including risk assessors) to iteratively develop a product that works well for its intended audience.

### Harmonization, Standardization, and Inter-Operability of WGS Techniques

The different procedures through which WGS data are generated and analyzed have a strong influence on the results. For example, the choice of different sequencing platforms introduces systematic biases that have an impact on the inferred phylogenies (Kaas et al., 2014). With foodborne AMR pathogens easily traversing jurisdictions or countries, it can be problematic when different laboratories use different procedures and do not arrive at identical conclusions (Pightling et al., 2018). Efforts toward harmonization and standardization of WGS techniques are ongoing, for example under the Global Microbial Identifier (Moran-Gilad et al., 2015) and the PulseNet International initiatives (Nadon et al., 2017), but a consensus on methods, quality measures and thresholds for data generation and analysis of foodborne AMR pathogens has yet to be established (Lüth et al., 2018). In the absence of a current consensus, it is important to ensure all sequenced genomes are made publicly available where possible, or available privately across jurisdictions, as to allow researchers to repeat analyses within their pipelines or under their parameters. Standardization of epidemiological data (also referred to as ‘metadata’) accompanying WGS data is also critical to provide requisite contextual information necessary to any microbial risk assessment activity (Hill et al., 2017). The Genomic Epidemiology Ontology (GenEpiO) Consortium seeks a global standard for the metadata associated with WGS data, including laboratory, clinical and epidemiological data fields (e.g., strain names harmonized and compatible with previous classification schemes), as well as existing food categories (Griffiths et al., 2017). Data sharing and inter-operability (e.g., between laboratory and epidemiological databases) should also be addressed as key priorities to ensure the efficient use of WGS data (Pightling et al., 2018).

### Toward Validation and Improvement of WGS-Based Profiles Using Other Omics Techniques

An increasing number of GWAS have been published in recent years; yet a majority of the genetic markers identified are putative markers, and only a few of them have been validated. Therefore, our ability to predict the phenotypic behavior of foodborne AMR hazards using WGS genetic markers still appears weak at this stage. Substantial work is needed to validate the correlations between phenotypic and WGS profiles before QMRA models can be based on WGS data only. To move forward into this direction, GWAS studies have great potential to benefit from the addition of other omics tools including transcriptomics, proteomics and metabolomics that would make it possible to assess whether genes or genetic markers are actually expressed (Franz et al., 2016). Cocolin et al. (2018) envisioned a framework by which QMRA would move beyond taxonomic and genotypic hazard identification to a more functional approach based on the study of microbial behavior using a combination of omics (so called ‘multi-omics’) techniques (Cocolin et al., 2018).

Additionally, bacterial phenotypic profiles (e.g., growth and survival characteristics) are influenced by the microbiota present in food, food environments or gastrointestinal tracts of humans and animals. Den Besten et al. (2018) strongly encouraged the use of metagenomics to help characterize the microbiota (including endogenous and pathogenic flora) of food and food processing facilities, as well as their changes over time under conditions associated with food processing, preservation and storage (Den Besten et al., 2018). The idea would be to use metagenomics to move toward the next generation of predictive microbiology models (i.e., inferring a bacterial population’s size evolution based on the initial contamination and the food environment) that include predictions of the behavior of the ecosystem as a whole, instead of focusing on a single hazard of interest. This would lead to a substantial improvement in the accuracy of exposure assessment models (Den Besten et al., 2018). Metagenomics could also be used to describe how the hazard interacts with the rest of the microbiome in the human gastrointestinal tract, accounting for the presence of an indigenous microbiota that provide a certain degree of colonization resistance, and the absence of protective microbiota in case of dysbiosis (Coleman et al., 2018). It represents an opportunity to move toward a new ‘health triangle’ paradigm in dose–response modeling that would replace the more conventional ‘disease triangle’ approach (focused on host, pathogen and environment) (Coleman et al., 2018).

Yet, metagenomics approaches also come with new challenges. While WGS rely on ‘clean’ genome sequence analyses, metagenomics require ‘noisy’ sequencing efforts where pathogenic bacteria only represent a minute amount of the bacterial community in a sample, so enrichment or removal of DNA from other sources are needed to improve data quality; these procedures introduce significant bias in the interpretation of metagenomics analyses (McArthur and Tsang, 2017; Cocolin et al., 2018). Additionally, while the presence of genes can be detected via metagenomes annotation, current tools are unable to identify which microorganisms the genes originated from, which represents a major issue for QMRA hazard identification (Cocolin et al., 2018).

### QMRA of Emerging AMR Hazards

Foodborne bacteria are constantly evolving new mechanisms to adapt and survive the lethal or biostatic effects of antimicrobials (Sekyere and Asante, 2018). This is challenging for WGS-based QMRA modeling. Gene prediction and annotation performed in the hazard identification step relies on databases of known resistance genes. Similarly, the study of the degree of relatedness between isolates conducted as part of the exposure assessment step requires comparison with a reference database of isolates believed to be representative of the bacterial population at a given point in time. In short, the information provided by WGS analysis is only as good as the reference databases that are used to generate them. Because of this issue, for the purpose of risk assessment of emerging AMR hazards, it will be critical to maintain a certain level of surveillance of foodborne AMR hazards based on phenotypic AST. Observed changes in phenotypic AMR profiles indeed suggest that new AMR molecular mechanisms (e.g., new AMR genes) may be developing. This is best illustrated with the recent identification of the *mcr-1* gene (and soon later the *mcr-2, mcr-3, mcr-4*, and *mcr-5* genes), which codes for plasmid-based resistance to polymyxin in Gram-negative bacteria (polymyxin is one of the few available drugs for treating infections caused by carbapenem-resistant *Enterobacteriaceae*) (Sekyere and Asante, 2018). A novel plasmid-based resistance mechanism to polymyxin was suspected following the observation of a major increase in phenotypic colistin resistance under a routine surveillance project on AMR in commensal *E. coli* from food animals in China (Liu et al., 2016). Whole plasmid sequencing and further functional study of those isolates later revealed the emergence of *mcr-1* (Liu et al., 2016). The rapid analysis of publicly available sequence databases showed that *mcr-1* had actually already spread to most continents (Skov and Monnet, 2016), and subsequent phylogenetic analyses demonstrated that all *mcr-1* elements in circulation descended from the same initial mobilization of *mcr-1* in the mid-2000s, followed by a marked demographic expansion (Wang et al., 2018). This example illustrates the capacity offered by WGS to query old genomes for newly discovered genes and determine whether those genes were present in historical samples. Surveillance based on phenotypic AST only would not have the ability to retrospectively assess the carriage of known AMR determinants. However, WGS-based AMR profiling may introduce a delay in the detection of AMR genes that can compromise the ability to assess the risk associated with emerging AMR hazards. Real-time update and curation of WGS reference databases, together with maintenance of a certain level of AMR surveillance based on phenotypic AST, will therefore be critical for AMR risk assessment purposes.

## Conclusion

The integration of WGS data into foodborne AMR QMRA modeling offers the opportunity to move toward the next-generation of AMR risk assessment. Instead of considering all hazard strains as equally able to cause disease, WGS can substantially improve the hazard identification by focusing on those strains of highest public health relevance and/or stratifying the hazards into subsets of similar genetic profiles that are expected to behave similarly. The distribution of QMRA model parameters, including growth/survival and pathogenicity/virulence can be adjusted accordingly, making it possible to capture the variability in the strains of interest while decreasing the uncertainty in some model input parameters. The high discriminatory power of WGS also offers an opportunity to improve the exposure assessment by analyzing the degree of relatedness between bacterial populations and AMR profiles found at successive stages of the food chain. Overall, WGS can contribute to substantial improvements to the accuracy of QMRA models, and should be considered in any future revision of the Codex Alimentarius Guidelines for Risk Analysis of Foodborne AMR. WGS data, however, also introduce new sources of uncertainty, especially related to the thresholds of relatedness to be used and the degree of correlation between genotypic and phenotypic profiles. The latter could be improved using a functional approach of microbial behavior based on a combination of omics techniques.

## Author Contributions

LC wrote the initial draft of the manuscript. PB, CC, BC, AF, BH, SM, EP, RR-S, ET, and BS provided substantial comments and edits to improve the text and figures and prepare the final version of the article.

## Conflict of Interest Statement

The authors declare that the research was conducted in the absence of any commercial or financial relationships that could be construed as a potential conflict of interest.

## Footnotes

1 https://lfz.corefacility.ca/sistr-app/

2 http://www.genomicepidemiology.org/

## References

[B1] Alikhan N. Zhou Z. Sergeant M. J. Achtman M. (2018). A genomic overview of the population structure of *Salmonella*. *PLoS Genet.* 14:e1007261. 10.1371/journal.pgen.1007261 29621240 PMC5886390

[B2] Allard M. W. Strain E. Melka D. Bunning K. Musser S. M. Brown E. W. (2016). Practical value of food pathogen traceability through building a whole-genome sequencing network and database. *J. Clin. Microbiol.* 54 1975–1983. 10.1128/JCM.00081-16 27008877 PMC4963501

[B3] Anjum M. F. (2015). Screening methods for the detection of antimicrobial resistance genes present in bacterial isolates and the microbiota. *Future Microbiol.* 10 317–320. 10.2217/fmb.15.2 25812454

[B4] Arts I. C. W. Weijenberg M. P. (2013). New training tools for new epidemiologists. *Environ. Mol. Mutagen.* 54 611–615. 10.1002/em.21793 23893701

[B5] Aun E. Brauer A. Kisand V. Tenson T. Remm M. (2018). A k-mer-based method for the identification of phenotype-associated genomic biomarkers and predicting phenotypes of sequenced bacteria. *PLoS Comput. Biol.* 14:e1006434. 10.1371/journal.pcbi.1006434 30346947 PMC6211763

[B6] Aung M. M. Chang Y. S. (2014). Traceability in a food supply chain: safety and quality perspectives. *Food Control* 39 172–184. 10.1016/j.foodcont.2013.11.007

[B7] Bartels M. D. Petersen A. Worning P. Nielsen J. B. Larner-Svensson H. Johansen H. K. (2014). Comparing whole-genome sequencing with Sanger sequencing for spa typing of Methicillin-Resistant *Staphylococcus aureus*. *J. Clin. Microbiol.* 52 4305–4308. 10.1128/JCM.01979-14 25297335 PMC4313303

[B8] Barza M. Travers K. (2002). Excess infections due to antimicrobial resistance: the “attributable fraction”. *Clin. Infect. Dis.* 34 S126–S130. 10.1086/340250 11988883

[B9] Bengtsson-Palme J. (2017). Antibiotic resistance in the food supply chain: where can sequencing and metagenomics aid risk assessment? *Curr. Opin. Food Sci*. 14 66–71. 10.1016/j.cofs.2017.01.010

[B10] Boerlin P. Reid-Smith R. J. (2008). Antimicrobial resistance: its emergence and transmission. *Anim. Health Res. Rev.* 9 115–126. 10.1017/S146625230800159X 19102787

[B11] Broaders E. Gahan C. G. M. Marchesi J. R. (2013). Mobile genetic elements of the human gastrointestinal tract: potential for spread of antibiotic resistance genes. *Gut Microbes* 4 271–280. 10.4161/gmic.24627 23651955 PMC3744512

[B12] Brul S. Bassett J. Cook P. Kathariou S. McClure P. Jasti P. R. (2012). ‘Omics’ technologies in quantitative microbial risk assessment. *Trends Food Sci. Technol.* 27 12–24. 10.1016/j.tifs.2012.04.004

[B13] Buchanan C. J. Webb A. L. Mutschall S. K. Kruczkiewicz P. Barker D. O. R. Hetman B. M. (2017). A genome-wide association study to identify diagnostic markers for human pathogenic *Campylobacter jejuni* strains. *Front. Microbiol.* 8:1224. 10.3389/fmicb.2017.01224 28713351 PMC5492696

[B14] Caffrey N. Invik J. Waldner C. L. Ramsay D. Checkley S. L. (2018). Risk assessments evaluating foodborne antimicrobial resistance in humans: a scoping review. *Microb. Risk Anal.* 11 31–46. 10.1016/j.mran.2018.08.002

[B15] Card R. M. Cawthraw S. A. Nunez-Garcia J. Ellis R. J. Kay G. Pallen M. J. (2017). An in vitro chicken gut model demonstrates transfer of a multidrug resistance plasmid from *Salmonella* to commensal *Escherichia coli*. *mBio* 8:e00777-17. 10.1128/mBio.00777-17 28720731 PMC5516254

[B16] Carrasco E. Morales-Rueda A. García-Gimeno R. M. (2012). Cross-contamination and recontamination by *Salmonella* in foods: a review. *Food Res. Int.* 45 545–556. 10.1016/j.foodres.2011.11.004

[B17] Carroll L. M. Wiedmann M. den Bakker H. Siler J. Warchocki S. Kent D. (2017). Whole-genome sequencing of drug-resistant *Salmonella enterica* isolated from dairy cattle and humans in New York and Washington states reveals source and geographic associations. *Appl. Environ. Microbiol.* 83:e00140-17. 10.1128/AEM.00140-17 28389536 PMC5452826

[B18] Chapman B. Pintar K. Smith B. A. (2018). Multi-exposure pathway model to compare *Escherichia coli* O157 risks and interventions. *Risk Anal.* 38 392–409. 10.1111/risa.12826 28471504

[B19] Chen Y. Ross W. H. Gray M. J. Wiedmann M. Whiting R. C. Scott V. N. (2006). Attributing risk to *Listeria monocytogenes* subgroups: dose response in relation to genetic lineages. *J. Food Prot.* 69 335–344. 10.4315/0362-028X-69.2.335 16496574

[B20] Chen Y. Ross W. H. Whiting R. C. Van Stelten A. Nightingale K. K. Wiedmann M. (2011). Variation in *Listeria monocytogenes* dose responses in relation to subtypes encoding a full-length or truncated Internalin A. *Appl. Environ. Microbiol.* 77 1171–1180. 10.1128/AEM.01564-10 21169442 PMC3067222

[B21] Christoph L. Sarno E. Collineau L. Meile L. Stärk K. D. C. Stephan R. (2018). Consumer exposure to antimicrobial resistant bacteria from food at Swiss retail level. *Front. Microbiol.* 9:362. 10.3389/fmicb.2018.00362 29559960 PMC5845543

[B22] Cocolin L. Mataragas M. Bourdichon F. Doulgeraki A. Pilet M. Jagadeesan B. (2018). Next generation microbiological risk assessment meta-omics: the next need for integration. *Int. J. Food Microbiol.* 287 10–17. 10.1016/j.ijfoodmicro.2017.11.008 29157743

[B23] Codex Alimentarius (1999). *Principles and Guidelines for the Conduct of Microbiological Risk Assessment. CAC/GL-30.* Available at: http://www.fao.org/3/y1579e/y1579e05.htm

[B24] Codex Alimentarius (2011). Guidelines for risk analysis of foodborne antimicrobial resistance. *CAC-GL* 77 1–29. 10.4315/JFP-22-038 35723548

[B25] Coleman M. Elkins C. Gutting B. Mongodin E. Solano-Aguilar G. Walls I. (2018). Microbiota and dose response: evolving paradigm of health triangle. *Risk Anal.* 38 2013–2028. 10.1111/risa.13121 29900563

[B26] Cosentino S. Voldby Larsen M. Møller Aarestrup F. Lund O. (2013). PathogenFinder - Distinguishing friend from foe using bacterial whole genome sequence data. *PLoS One* 8:e77302. 10.1371/journal.pone.0077302 24204795 PMC3810466

[B27] De Been M. Lanza V. F. de Toro M. Scharringa J. Dohmen W. Du Y. (2014). Dissemination of cephalosporin resistance genes between *Escherichia coli* strains from farm animals and humans by specific plasmid lineages. *PLoS Genet.* 10:e1004776. 10.1371/journal.pgen.1004776 25522320 PMC4270446

[B28] De Lamballerie X. (2009). Essential veterinary education in modern molecular tools for the detection of disease: what veterinarians will need to know about genomics and molecular biology and diagnostics (including bioterrorist weapons) in 2025. *Rev. Sci. Tech.* 28 657–662. 20128476 10.20506/rst.28.2.1909

[B29] Dearlove B. L. Cody A. J. Pascoe B. Méric G. Wilson D. J. Sheppard S. K. (2016). Rapid host switching in generalist *Campylobacter* strains erodes the signal for tracing human infections. *ISME J.* 10 721–729. 10.1038/ismej.2015.149 26305157 PMC4677457

[B30] Deckert A. Agunos A. Avery B. Carson C. Daignault D. Finley R. (2015). CIPARS: a One-Health approach to antimicrobial resistance surveillance. *Online J. Public Health Inform.* 7:e68 10.5210/ojphi.v7i1.5734

[B31] Den Besten H. M. W. Amézquita A. Bover-Cid S. Dagnas S. Ellouze M. Guillou S. (2018). Next generation of microbiological risk assessment: potential of omics data for exposure assessment. *Int. J. Food Microbiol.* 287 18–27. 10.1016/j.ijfoodmicro.2017.10.006 29032838

[B32] Dorado-García A. Mevius D. J. Jacobs J. J. H. Van Geijlswijk I. M. Mouton J. W. Wagenaar J. A. (2016). Quantitative assessment of antimicrobial resistance in livestock during the course of a nationwide antimicrobial use reduction in the Netherlands. *J. Antimicrob. Chemother.* 71 3607–3619. 10.1093/jac/dkw308 27585970

[B33] Dutil L. Irwin R. Finley R. Ng L. K. Avery B. Boerlin P. (2010). Ceftiofur resistance in *Salmonella enterica* serovar Heidelberg from chicken meat and humans, Canada. *Emerg. Infect. Dis.* 16 48–54. 10.3201/eid1601.090729 20031042 PMC2874360

[B34] Earle S. G. Wu C.-H. Charlesworth J. Stoesser N. Gordon N. C. Walker T. M. (2016). Identifying lineage effects when controlling for population structure improves power in bacterial association studies. *Nat. Microbiol.* 1:16041. 10.1038/nmicrobiol.2016.41 27572646 PMC5049680

[B35] Edirmanasinghe R. Finley R. Parmley E. J. Avery B. P. Carson C. Bekal S. (2017). A whole-genome sequencing approach to study cefoxitin-resistant *Salmonella enterica* serovar Heidelberg isolates from various sources. *Antimicrob. Agents Chemother.* 61:e01919-16. 10.1128/AAC.01919-16 28137797 PMC5365727

[B36] European Food Safety Authority [EFSA] (2008). Foodborne antimicrobial resistance as a biological hazard - scientific opinion of the panel on biological hazards. *EFSA J.* 6 765–787. 10.2903/j.efsa.2008.765 PMC10193654 37213857

[B37] European Food Safety Authority [EFSA] (2013). Scientific Opinion on the evaluation of molecular typing methods for major food-borne microbiological hazards and their use for attribution modelling, outbreak investigation and scanning surveillance: part 1 (evaluation of methods and applications). *EFSA J.* 11:3502 10.2903/j.efsa.2013.3502

[B38] European Food Safety Authority [EFSA] (2018). Outcome of EC/EFSA questionnaire (2016) on use of Whole Genome Sequencing (WGS) for food- and waterborne pathogens isolated from animals, food, feed and related environmental samples in EU/EFTA countries. *EFSA Support. Publ.* 15:1432E 10.2903/sp.efsa.2018.EN-1432

[B39] Falush D. (2016). Bacterial genomics: microbial GWAS coming of age. *Nat. Microbiol.* 1:16059. 10.1038/nmicrobiol.2016.59 27572652

[B40] FAO/WHO (2002). *Risk Assessments of Salmonella in Eggs and Broiler Chickens. Microbiological Risk Assessment Series. (2), Technical Report.* Geneva: WHO.

[B41] Fegan N. Jenson I. (2018). The role of meat in foodborne disease: is there a coming revolution in risk assessment and management? *Meat Sci.* 144 22–29. 10.1016/j.meatsci.2018.04.018 29716760

[B42] Food and Drug Administration (2018). *National Antimicrobial Resistance Monitoring System (NARMS) Webpage.* Available at: https://www.fda.gov/animalveterinary/safetyhealth/antimicrobialresistance/nationalantimicrobialresistancemonitoringsystem/ucm059089.htm (accessed December 21, 2018).

[B43] Franz E. Gras L. M. Dallman T. (2016). Significance of whole genome sequencing for surveillance, source attribution and microbial risk assessment of foodborne pathogens. *Curr. Opin. Food Sci.* 8 74–79. 10.1016/j.cofs.2016.04.004

[B44] Fritsch L. Felten A. Palma F. Mariet J. Radomski N. Mistou M. (2018a). Insights from genome-wide approaches to identify variants associated to phenotypes at pan-genome scale: application to *L. monocytogenes*’ ability to grow in cold conditions. *Int. J. Food Microbiol.* 291 181–188. 10.1016/j.ijfoodmicro.2018.11.028 30530095

[B45] Fritsch L. Guillier L. Augustin J. (2018b). Next generation quantitative microbiological risk assessment: refinement of the cold smoked salmon-related listeriosis risk model by integrating genomic data. *Microb. Risk Anal.* 10 20–27. 10.1016/j.mran.2018.06.003

[B46] Gordon N. C. Price J. R. Cole K. Everitt R. Morgan M. Finney J. (2014). Prediction of *Staphylococcus aureus* antimicrobial resistance by whole-genome sequencing. *J. Clin. Microbiol.* 52 1182–1191. 10.1128/JCM.03117-13 24501024 PMC3993491

[B47] Griffiths E. Dooley D. Graham M. Van Domselaar G. Brinkman F. S. L. Hsiao W. W. L. (2017). Context is everything: harmonization of critical food microbiology descriptors and metadata for improved food safety and surveillance. *Front. Microbiol.* 8:1068. 10.3389/fmicb.2017.01068 28694792 PMC5483436

[B48] Haagsma J. A. Geenen P. L. Ethelberg S. Fetsch A. Hansdotter F. Jansen A. (2013). Community incidence of pathogen-specific gastroenteritis: reconstructing the surveillance pyramid for seven pathogens in seven European Union member states. *Epidemiol. Infect.* 141 1625–1639. 10.1017/S0950268812002166 23013659 PMC9151593

[B49] Haddad N. Johnson N. Kathariou S. Métris A. Phister T. Pielaat A. (2018). Next generation microbiological risk assessment—Potential of omics data for hazard characterisation. *Int. J. Food Microbiol.* 287 28–39. 10.1016/j.ijfoodmicro.2018.04.015 29703417

[B50] Han J. Lynne A. M. David D. E. Tang H. Xu J. Nayak R. (2012). DNA sequence analysis of plasmids from multidrug resistant *Salmonella enterica* serotype Heidelberg isolates. *PLoS One* 7:e51160. 10.1371/journal.pone.0051160 23251446 PMC3519518

[B51] Heather J. M. Chain B. (2016). The sequence of sequencers: the history of sequencing DNA. *Genomics* 107 1–8. 10.1016/j.ygeno.2015.11.003 26554401 PMC4727787

[B52] Henri C. Leekitcharoenphon P. Carleton H. A. Radomski N. Kaas R. S. Mariet J. (2017). An assessment of different genomic approaches for inferring phylogeny of *Listeria monocytogenes*. *Front. Microbiol.* 8:2351. 10.3389/fmicb.2017.02351 29238330 PMC5712588

[B53] Hetman B. M. Mutschall S. K. Thomas J. E. Gannon V. P. J. Clark C. G. Pollari F. (2017). The EpiQuant framework for computing the epidemiological concordance of microbial subtyping data. *J. Clin. Microbiol.* 55:JCM.01945-16. 10.1128/JCM.01945-16 28202797 PMC5405252

[B54] Hill A. A. Crotta M. Wall B. Good L. O’Brien S. J. Guitian J. (2017). Towards an integrated food safety surveillance system: a simulation study to explore the potential of combining genomic and epidemiological metadata. *R. Soc. Open Sci.* 4:160721. 10.1098/rsos.160721 28405360 PMC5383817

[B55] Hingston P. Chen J. Dhillon B. K. Laing C. Bertelli C. Gannon V. (2017). Genotypes associated with *Listeria monocytogenes* isolates displaying impaired or enhanced tolerances to cold, salt, acid, or desiccation stress. *Front. Microbiol.* 8:369 10.3389/fmicb.2017.00369 PMC5340757 28337186

[B56] Holmes A. H. Moore L. S. P. Sundsfjord A. Steinbakk M. Regmi S. Karkey A. (2016). Understanding the mechanisms and drivers of antimicrobial resistance. *Lancet* 387 176–187. 10.1016/S0140-6736(15)00473-0 26603922

[B57] Huddleston J. R. (2014). Horizontal gene transfer in the human gastrointestinal tract: potential spread of antibiotic resistance genes. *Infect. Drug Resist.* 7 167–176. 10.2147/IDR.S48820 25018641 PMC4073975

[B58] Hunt M. Mather A. E. Sánchez-Busó L. Page A. J. Parkhill J. Keane J. A. (2017). ARIBA: rapid antimicrobial resistance genotyping directly from sequencing reads. *Microb. Genomics* 3:e000131. 10.1099/mgen.0.000131 29177089 PMC5695208

[B59] Institute of Medicine (1989). *Human Health Risks with the Subtherapeutic use of Penicillin or Tetracyclines in Animal Feed.* Washington, DC: The National Academies Press.

[B60] Jia B. Raphenya A. R. Alcock B. Waglechner N. Guo P. Tsang K. K. (2016). CARD 2017: expansion and model-centric curation of the comprehensive antibiotic resistance database. *Nucleic Acids Res.* 45 D566–D573. 10.1093/nar/gkw1004 27789705 PMC5210516

[B61] Joensen K. G. Scheutz F. Lund O. Hasman H. Kaas R. S. Nielsen E. M. (2014). Real-time whole-genome sequencing for routine typing, surveillance, and outbreak detection of verotoxigenic *Escherichia coli*. *J. Clin. Microbiol.* 52 1501–1510. 10.1128/JCM.02562-14 24574290 PMC3993690

[B62] Jones T. F. Ingram L. A. Cieslak P. R. Vugia D. J. Tobin-D’Angelo M. Hurd S. (2008). Salmonellosis outcomes differ substantially by serotype. *J. Infect. Dis.* 198 109–114. 10.1086/588823 18462137

[B63] Kaas R. S. Leekitcharoenphon P. Aarestrup F. M. Lund O. (2014). Solving the problem of comparing whole bacterial genomes across different sequencing platforms. *PLoS One* 9:e104984. 10.1371/journal.pone.0104984 25110940 PMC4128722

[B64] Lakin S. M. Dean C. Noyes N. R. Dettenwanger A. Ross A. S. Doster E. (2017). MEGARes: an antimicrobial resistance database for high throughput sequencing. *Nucleic Acids Res.* 45 D574–D580. 10.1093/nar/gkw1009 27899569 PMC5210519

[B65] Le Devendec L. Jouy E. Kempf I. (2018). Evaluation of resistance gene transfer from heat-treated *Escherichia coli*. *Int. J. Food Microbiol.* 270 39–43. 10.1016/j.ijfoodmicro.2018.02.019 29477666

[B66] Leekitcharoenphon P. Garcia-Graells C. Botteldoorn N. Dierick K. Kempf I. Olkkola S. (2018). Comparative genomics of quinolone-resistant and susceptible *Campylobacter jejuni* of poultry origin from major poultry producing European countries (GENCAMP). *EFSA Support. Publ.* 15:1398E 10.2903/sp.efsa.2018.EN-1398

[B67] Lees J. A. Bentley S. D. (2016). Bacterial GWAS: not just gilding the lily. *Nat. Rev. Microbiol.* 14:406. 10.1038/nrmicro.2016.82 27241043

[B68] Lester C. H. Frimodt-Møller N. Sørensen T. L. Monnet D. L. Hammerum A. M. (2006). In vivo transfer of the vanA resistance gene from an *Enterococcus faecium* isolate of animal origin to an *E. faecium* isolate of human origin in the intestines of human volunteers. *Antimicrob. Agents Chemother.* 50 596–599. 10.1128/AAC.50.2.596-599.2006 16436715 PMC1366888

[B69] Liu Y. Wang Y. Walsh T. R. Yi L. Zhang R. Spencer J. (2016). Emergence of plasmid-mediated colistin resistance mechanism MCR-1 in animals and human beings in China: a microbiological and molecular biological study. *Lancet Infect. Dis.* 16 161–168. 10.1016/S1473-3099(15)00424-7 26603172

[B70] Lüth S. Kleta S. Al Dahouk S. (2018). Whole genome sequencing as a typing tool for foodborne pathogens like *Listeria monocytogenes* – The way towards global harmonisation and data exchange. *Trends Food Sci. Technol.* 73 67–75. 10.1016/j.tifs.2018.01.008

[B71] Madec J. Haenni M. (2018). Antimicrobial resistance plasmid reservoir in food and food-producing animals. *Plasmid* 99 72–81. 10.1016/j.plasmid.2018.09.001 30194944

[B72] Maiden M. C. Jansen van Rensburg M. J. Bray J. E. Earle S. G. Ford S. A. Jolley K. A. (2013). MLST revisited: the gene-by-gene approach to bacterial genomics. *Nat. Rev. Microbiol.* 11 728–736. 10.1038/nrmicro3093 23979428 PMC3980634

[B73] Majowicz S. E. Scallan E. Jones-Bitton A. Sargeant J. M. Stapleton J. Angulo F. J. (2014). Global incidence of human shiga toxin–producing *Escherichia coli* infections and deaths: a systematic review and knowledge synthesis. *Foodborne Pathog. Dis.* 11 447–455. 10.1089/fpd.2013.1704 24750096 PMC4607253

[B74] Mandal R. K. Kwon Y. M. (2017). Global screening of *Salmonella enterica* serovar Typhimurium genes for desiccation survival. *Front. Microbiol.* 8:1723. 10.3389/fmicb.2017.01723 28943871 PMC5596212

[B75] McArthur A. G. Tsang K. K. (2017). Antimicrobial resistance surveillance in the genomic age. *Ann. N. Y. Acad. Sci.* 1388 78–91. 10.1111/nyas.13289 27875856

[B76] McEwen S. A. (2012). Quantitative human health risk assessments of antimicrobial use in animals and selection of resistance: a review of publicly available reports. *Rev. Sci. Tech.* 31 261–276. 22849281 10.20506/rst.31.1.2116

[B77] Mo S. S. Sunde M. Ilag H. K. Langsrud S. Heird E. (2017). Transfer potential of plasmids conferring extended-spectrum-cephalosporin resistance in *Escherichia coli* from poultry. *Appl. Environ. Microbiol.* 83:e00654-17. 10.1128/AEM.00654-17 28411217 PMC5452821

[B78] Mook P. Gardiner D. Verlander N. Q. McCormick J. Usdin M. Crook P. (2018). Operational burden of implementing *Salmonella* Enteritidis and Typhimurium cluster detection using whole genome sequencing surveillance data in England: a retrospective assessment. *Epidemiol. Infect.* 146 1452–1460. 10.1017/S0950268818001589 29961436 PMC9133683

[B79] Moran-Gilad J. Sintchenko V. Pedersen S. K. Wolfgang W. J. Pettengill J. Strain E. (2015). Proficiency testing for bacterial whole genome sequencing: an end-user survey of current capabilities, requirements and priorities. *BMC Infect. Dis.* 15:174. 10.1186/s12879-015-0902-3 25887164 PMC4392855

[B80] Muloi D. Ward M. J. Pedersen A. B. Fèvre E. M. Woolhouse M. E. J. Van Bunnik B. A. D. (2018). Are food animals responsible for transfer of antimicrobial-resistant *Escherichia coli* or their resistance determinants to human populations? A systematic review. *Foodborne Pathog. Dis.* 15 467–474. 10.1089/fpd.2017.2411 29708778 PMC6103250

[B81] Munk P. Knudsen B. E. Lukjacenko O. Duarte A. S. R. Van Gompel L. Luiken R. E. C. (2018). Abundance and diversity of the faecal resistome in slaughter pigs and broilers in nine European countries. *Nat. Microbiol.* 3 898–908. 10.1038/s41564-018-0192-9 30038308

[B82] Murphy C. P. Carson C. Smith B. A. Chapman B. Marrotte J. McCann M. (2018). Factors potentially linked with the occurrence of antimicrobial resistance in selected bacteria from cattle, chickens and pigs: a scoping review of publications for use in modelling of antimicrobial resistance (IAM.AMR Project). *Zoonoses Public Health* 65 957–971. 10.1111/zph.12515 30187682

[B83] Murray C. J. L. (1994). Quantifying the burden of disease: the technical basis for disability-adjusted life years. *Bull. World Health Organ.* 72 429–445. 8062401 PMC2486718

[B84] Nadon C. Van Walle I. Gerner-Smidt P. Campos J. Chinen I. Concepcion-Acevedo J. (2017). PulseNet international: vision for the implementation of whole genome sequencing (WGS) for global foodborne disease surveillance. *Euro Surveill.* 22:30544. 10.2807/1560-7917.ES.2017.22.23.30544 28662764 PMC5479977

[B85] Nastasijevic I. Milanov D. Velebit B. Djordjevic V. Swift C. Painset A. (2017). Tracking of *Listeria monocytogenes* in meat establishment using whole genome sequencing as a food safety management tool: a proof of concept. *Int. J. Food Microbiol.* 257 157–164. 10.1016/j.ijfoodmicro.2017.06.015 28666130

[B86] Nauta M. Van D. F. Havelaar A. (2005). A poultry-processing model for quantitative microbiological risk assessment. *Risk Anal.* 25 85–98. 10.1111/j.0272-4332.2005.00569.x 15787759

[B87] Neuert S. Nair S. Day M. R. Doumith M. Ashton P. M. Mellor K. C. (2018). Prediction of phenotypic antimicrobial resistance profiles from whole genome sequences of non-typhoidal *Salmonella enterica*. *Front. Microbiol.* 9:592. 10.3389/fmicb.2018.00592 29636749 PMC5880904

[B88] Newell D. G. Koopmans M. Verhoef L. Duizer E. Aidara-Kane A. Sprong H. (2010). Food-borne diseases - The challenges of 20 years ago still persist while new ones continue to emerge. *Int. J. Food Microbiol.* 139 S3–S15. 10.1016/j.ijfoodmicro.2010.01.021 20153070 PMC7132498

[B89] Nielsen E. M. Björkman J. T. Kiil K. Grant K. Dallman T. Painset A. (2017). Closing gaps for performing a risk assessment on *Listeria monocytogenes* in ready-to-eat (RTE) foods: activity 3, the comparison of isolates from different compartments along the food chain, and from humans using whole genome sequencing (WGS) analysis. *EFSA Support. Publ.* 14:1151E.

[B90] O’Neill J. (2014). *Antimicrobial Resistance: Tackling a Crisis for the Health and Wealth of Nations. Review on Antimicrobial Resistance.* Available at: https://amr-review.org/sites/default/files/AMR Review Paper - Tackling a crisis for the health and wealth of nations_1.pdf (accessed June 19, 2018).

[B91] Oniciuc E. A. Likotrafiti E. Alvarez-Molina A. Prieto M. Santos J. A. Alvarez-Ordóñez A. (2018). The present and future of Whole Genome Sequencing (WGS) and Whole Metagenome Sequencing (WMS) for surveillance of antimicrobial resistant microorganisms and antimicrobial resistance genes across the food chain. *Genes* 9:E268. 10.3390/genes9050268 29789467 PMC5977208

[B92] Orlek A. Stoesser N. Anjum M. F. Doumith M. Ellington M. J. Peto T. (2017). Plasmid classification in an era of whole-genome sequencing: application in studies of antibiotic resistance epidemiology. *Front. Microbiol.* 8:182. 10.3389/fmicb.2017.00182 28232822 PMC5299020

[B93] Oscar T. P. (2009). General regression neural network and Monte Carlo simulation model for survival and growth of *Salmonella* on raw chicken skin as a function of serotype, temperature, and time for use in risk assessment. *J. Food Prot.* 72 2078–2087. 10.4315/0362-028X-72.10.2078 19833030

[B94] Otto S. J. G. Carson C. A. Finley R. L. Thomas M. K. Reid-Smith R. McEwen S. A. (2014). Estimating the number of human cases of ceftiofur-resistant *Salmonella enterica* serovar Heidelberg in Quebec and Ontario, Canada. *Clin. Infect. Dis.* 59 1281–1290. 10.1093/cid/ciu496 24982036

[B95] Parisi A. Crump J. A. Glass K. Howden B. P. Furuya-Kanamori L. Vilkins S. (2018). Health outcomes from multidrug-resistant *Salmonella* infections in high-income countries: a systematic review and meta-analysis. *Foodborne Pathog. Dis.* 15 428–436. 10.1089/fpd.2017.2403 29624414

[B96] Partridge S. R. Kwong S. M. Firth N. Jensen S. O. (2018). Mobile genetic elements associated with antimicrobial resistance. *Clin. Microbiol. Rev.* 31:e00088-17. 10.1128/CMR.00088-17 30068738 PMC6148190

[B97] Pearce M. E. Alikhan N. Dallman T. J. Zhou Z. Grant K. Maiden M. C. J. (2018). Comparative analysis of core genome MLST and SNP typing within a European *Salmonella* serovar Enteritidis outbreak. *Int. J. Food Microbiol.* 274 1–11. 10.1016/j.ijfoodmicro.2018.02.023 29574242 PMC5899760

[B98] Pielaat A. Boer M. P. Wijnands L. M. van Hoek A. H. A. M. Bouw E. Barker G. C. (2015). First step in using molecular data for microbial food safety risk assessment; hazard identification of *Escherichia coli* O157:H7 by coupling genomic data with in vitro adherence to human epithelial cells. *Int. J. Food Microbiol.* 213 130–138. 10.1016/j.ijfoodmicro.2015.04.009 25910947 PMC4613885

[B99] Pightling A. W. Pettengill J. B. Luo Y. Baugher J. D. Rand H. Strain E. (2018). Interpreting whole-genome sequence analyses of foodborne bacteria for regulatory applications and outbreak investigations. *Front. Microbiol.* 9:1482. 10.3389/fmicb.2018.01482 30042741 PMC6048267

[B100] Pintar K. D. M. Thomas K. M. Christidis T. Otten A. Nesbitt A. Marshall B. (2017). A comparative exposure assessment of *Campylobacter* in Ontario, Canada. *Risk Anal.* 37 677–715. 10.1111/risa.12653 27641939

[B101] Pires S. M. Duarte A. S. Hald T. (2018). Source attribution and risk assessment of antimicrobial resistance. *Microbiol. Spectr.* 6:ARBA-0027-2017. 10.1128/microbiolspec.ARBA-0027-2017 29916343 PMC11633588

[B102] Poppe C. Martin L. C. Gyles C. L. Reid-Smith R. Boerlin P. McEwen S. A. (2005). Acquisition of resistance to extended-spectrum cephalosporins by *Salmonella enterica* subsp. *enterica* serovar Newport and *Escherichia coli* in the turkey poult intestinal tract. *Appl. Environ. Microbiol.* 71 1184–1192. 10.1128/AEM.71.3.1184-1192.2005 15746317 PMC1065184

[B103] Projahn M. von Tippelskirch P. Semmler T. Guenther S. Alter T. Roesler U. (2019). Contamination of chicken meat with extended-spectrum beta-lactamase producing- *Klebsiella pneumoniae* and *Escherichia coli* during scalding and defeathering of broiler carcasses. *Food Microbiol.* 77 185–191. 10.1016/j.fm.2018.09.010 30297049

[B104] Public Health Agency of Canada (2018). *FOODNET CANADA 2017 Stakeholder Update.* Available at: http://publications.gc.ca/collections/collection_2018/aspc-phac/HP37-29-2018-eng.pdf (accessed December 21, 2018).

[B105] Rahman S. M. A. Munther D. Fazil A. Smith B. Wu J. (2016). Unraveling the dose-response puzzle of *L. monocytogenes*: a mechanistic approach. *Infect. Dis. Model.* 1 101–114. 10.1016/j.idm.2016.09.001 29928724 PMC5963320

[B106] Rahman S. M. A. Munther D. Fazil A. Smith B. Wu J. (2018). Advancing risk assessment: mechanistic dose-response modelling of *Listeria monocytogenes* infection in human populations. *R. Soc. Open Sci.* 5:180343. 10.1098/rsos.180343 30225020 PMC6124125

[B107] Rantsiou K. Kathariou S. Winkler A. Skandamis P. Saint-Cyr M. Rouzeau-Szynalski K. (2018). Next generation microbiological risk assessment opportunities of whole genome sequencing (WGS) for foodborne pathogen surveillance, source tracking and risk assessment. *Int. J. Food Microbiol.* 287 3–9. 10.1016/j.ijfoodmicro.2017.11.007 29246458

[B108] Ribeiro-Gonçalves B. Francisco A. P. Vaz C. Ramirez M. Carriço J. A. (2016). PHYLOViZ Online: web-based tool for visualization, phylogenetic inference, analysis and sharing of minimum spanning trees. *Nucleic Acids Res.* 44 W246–W251. 10.1093/nar/gkw359 27131357 PMC4987911

[B109] Robertson J. Nash J. H. E. (2018). MOB-suite: software tools for clustering, reconstruction and typing of plasmids from draft assemblies. *Microb. Genomics* 4:e000206. 10.1099/mgen.0.000206 30052170 PMC6159552

[B110] Roer L. Hendriksen R. S. Leekitcharoenphon P. Lukjancenko O. Kaas R. S. Hasman H. (2016). Is the evolution of *Salmonella enterica* subsp. enterica linked to restriction-modification systems? *mSystems* 1:e00009-16. 10.1128/mSystems.00009-16 27822532 PMC5069764

[B111] Ronholm J. Nasheri N. Petronella N. Pagotto F. (2016). Navigating microbiological food safety in the era of whole-genome sequencing. *Clin. Microbiol. Rev.* 29 837–857. 10.1128/CMR.00056-16 27559074 PMC5010751

[B112] Rowe W. Baker K. S. Verner-Jeffreys D. Baker-Austin C. Ryan J. J. Maskell D. (2015). Search engine for antimicrobial resistance: a cloud compatible pipeline and web interface for rapidly detecting antimicrobial resistance genes directly from sequence data. *PLoS One* 10:e0133492. 10.1371/journal.pone.0133492 26197475 PMC4510569

[B113] Rozov R. Brown Kav A. Bogumil D. Shterzer N. Halperin E. Mizrahi I. (2017). Recycler: an algorithm for detecting plasmids from de novo assembly graphs. *Bioinformatics* 33 475–482. 10.1093/bioinformatics/btw651 28003256 PMC5408804

[B114] Sanaa M. Pouillot R. Vega F. G. Strain E. Van Doren J. M. (2019). GenomeGraphR: a user-friendly open-source web application for foodborne pathogen whole genome sequencing data integration, analysis, and visualization. *PLoS One* 14:e0213039. 10.1371/journal.pone.0213039 30818354 PMC6394949

[B115] Schürch A. C. Arredondo-Alonso S. Willems R. J. L. Goering R. V. (2018). Whole genome sequencing options for bacterial strain typing and epidemiologic analysis based on single nucleotide polymorphism versus gene-by-gene–based approaches. *Clin. Microbiol. Infect.* 24 350–354. 10.1016/j.cmi.2017.12.016 29309930

[B116] Sekyere J. O. Asante J. (2018). Emerging mechanisms of antimicrobial resistance in bacteria and fungi: advances in the era of genomics. *Future Microbiol.* 13 241–262. 10.2217/fmb-2017-0172 29319341

[B117] Shelburne S. A. Kim J. Munita J. M. Sahasrabhojane P. Shields R. K. Press E. G. (2017). Whole-genome sequencing accurately identifies resistance to extended-spectrum ß-lactams for major gram-negative bacterial pathogens. *Clin. Infect. Dis.* 65 738–745. 10.1093/cid/cix417 28472260 PMC5850535

[B118] Sheppard S. K. Didelot X. Meric G. Torralbo A. Jolley K. A. Kelly D. J. (2013). Genome-wide association study identifies vitamin B5 biosynthesis as a host specificity factor in *Campylobacter*. *Proc. Natl. Acad. Sci. U.S.A.* 110 11923–11927. 10.1073/pnas.1305559110 23818615 PMC3718156

[B119] Singh G. Vajpayee P. Rani N. Amoah I. D. Stenström T. A. Shanker R. (2016). Exploring the potential reservoirs of non specific TEM beta lactamase (blaTEM) gene in the Indo-Gangetic region: a risk assessment approach to predict health hazards. *J. Hazard. Mater.* 314 121–128. 10.1016/j.jhazmat.2016.04.036 27111425

[B120] Skov R. L. Monnet D. L. (2016). Plasmid-mediated colistin resistance (mcr-1 gene): three months later, the story unfolds. *Euro Surveill.* 21:30155. 10.2807/1560-7917.ES.2016.21.9.30155 26967914

[B121] Smith R. Coast J. (2013). The true cost of antimicrobial resistance. *BMJ* 346:f1493. 10.1136/bmj.f1493 23479660

[B122] Stoesser N. Batty E. M. Eyre D. W. Morgan M. Wyllie D. H. Del Ojo Elias C. (2013). Predicting antimicrobial susceptibilities for *Escherichia coli* and *Klebsiella pneumoniae* isolates using whole genomic sequence data. *J. Antimicrob. Chemother.* 68 2234–2244. 10.1093/jac/dkt180 23722448 PMC3772739

[B123] Taboada E. N. Graham M. R. Carriço J. A. Van Domselaar G. (2017). Food safety in the age of next generation sequencing, bioinformatics, and open data access. *Front. Microbiol.* 8:909. 10.3389/fmicb.2017.00909 28588568 PMC5440521

[B124] Tang K. L. Caffrey N. P. Nóbrega D. B. Cork S. C. Ronksley P. E. Barkema H. W. (2017). Restricting the use of antibiotics in food-producing animals and its associations with antibiotic resistance in food-producing animals and human beings: a systematic review and meta-analysis. *Lancet Planet. Health* 1 e316–e327. 10.1016/S2542-5196(17)30141-9 29387833 PMC5785333

[B125] Tassios P. T. Moran-Gilad J. (2018). Bacterial next generation sequencing (NGS) made easy. *Clin. Microbiol. Infect.* 24 332–334. 10.1016/j.cmi.2018.03.001 29548687

[B126] Teunis P. F. M. Kasuga F. Fazil A. Ogden I. D. Rotariu O. Strachan N. J. C. (2010). Dose–response modeling of *Salmonella* using outbreak data. *Int. J. Food Microbiol.* 144 243–249. 10.1016/j.ijfoodmicro.2010.09.026 21036411

[B127] Teunis P. F. M. van der Heijden O. G. van der Giessen J. W. B. Havelaar A. H. (1996). *The Dose Response Relation in Human Volunteers for Gastrointestinal Pathogens. Report nr 284 550 002.* Bilthoven: National Institute for Public Health and the Environment.

[B128] Thépault A. Méric G. Rivoal K. Pascoe B. Mageiros L. Touzain F. (2017). Genome-wide identification of host-segregating epidemiological markers for source attribution in *Campylobacter jejuni*. *Appl. Environ. Microbiol.* 83 e03085-16. 10.1128/AEM.03085-16 28115376 PMC5359498

[B129] Tyson G. H. Zhao S. Li C. Ayers S. Sabo J. L. Lam C. (2017). Establishing genotypic cutoff values to measure antimicrobial resistance in *Salmonella*. *Antimicrob. Agents Chemother.* 61:e02140-16. 10.1128/AAC.02140-16 27993845 PMC5328538

[B130] Van Belkum A. Tassios P. T. Dijkshoorn L. Haeggman S. Cookson B. Fry N. K. (2007). Guidelines for the validation and application of typing methods for use in bacterial epidemiology. *Clin. Microbiol. Infect.* 13 1–46. 10.1111/j.1469-0691.2007.01786.x 17716294

[B131] Verraes C. Van Boxstael S. Van Meervenne E. Van Coillie E. Butaye P. Catry B. (2013). Antimicrobial resistance in the food chain: a review. *Int. J. Environ. Res. Public Health* 10 2643–2669. 10.3390/ijerph10072643 23812024 PMC3734448

[B132] Vohra P. Bugarel M. Turner F. Loneragan G. H. Hope J. C. Hopkins J. (2018). Quantifying the survival of multiple *Salmonella enterica* serovars in vivo via massively parallel whole-genome sequencing to predict zoonotic risk. *Appl. Environ. Microbiol.* 84:e02262-17. 10.1128/AEM.02262-17 29180370 PMC5795071

[B133] Volkova V. V. Lu Z. Lanzas C. Scott H. M. Gröhn Y. T. (2013). Modelling dynamics of plasmid-gene mediated antimicrobial resistance in enteric bacteria using stochastic differential equations. *Sci. Rep.* 3:2463. 10.1038/srep02463 23982723 PMC3755285

[B134] Wales A. D. Davies R. H. (2015). Co-selection of resistance to antibiotics, biocides and heavy metals, and its relevance to foodborne pathogens. *Antibiotics* 4 567–604. 10.3390/antibiotics4040567 27025641 PMC4790313

[B135] Wang R. Van Dorp L. Shaw L. P. Bradley P. Wang Q. Wang X. (2018). The global distribution and spread of the mobilized colistin resistance gene mcr-1. *Nat. Commun.* 9:1179. 10.1038/s41467-018-03205-z 29563494 PMC5862964

[B136] Wijnands L. M. Teunis P. F. M. Kuijpers A. F. A. Delfgou-Van Asch E. H. M. Pielaat A. (2017). Quantification of *Salmonella* survival and infection in an *in vitro* model of the human intestinal tract as proxy for foodborne pathogens. *Front. Microbiol.* 8:1139. 10.3389/fmicb.2017.01139 28713334 PMC5491934

[B137] World Health Organization [WHO] (2014). *Antimicrobial Resistance: Global Report on Surveillance.* Available at: https://www.who.int/antimicrobial-resistance/publications/surveillancereport/en/ (accessed December 21, 2018).

[B138] World Health Organization [WHO] (2015a). *Global Action Plan on Antimicrobial Resistance. WHO Library Cataloguing-in-Publication Data.* Available at: https://www.who.int/antimicrobial-resistance/global-action-plan/en/ (accessed December 21 2018).

[B139] World Health Organization [WHO] (2015b). *WHO Estimates of the Global Burden of Foodborne Diseases: Foodborne Disease Burden Epidemiology Reference Group 2007-2015.* Geneva: WHO.

[B140] Yahara K. Méric G. Taylor A. J. de Vries S. P. W. Murray S. Pascoe B. (2017). Genome-wide association of functional traits linked with *Campylobacter jejuni* survival from farm to fork. *Environ. Microbiol.* 19 361–380. 10.1111/1462-2920.13628 27883255

[B141] Yoshida C. E. Kruczkiewicz P. Laing C. R. Lingohr E. J. Gannon V. P. Nash J. H. (2016). The *Salmonella* in silico typing resource (SISTR): an open web-accessible tool for rapidly typing and subtyping draft *Salmonella* genome assemblies. *PLoS One* 11:e0147101. 10.1371/journal.pone.0147101 26800248 PMC4723315

[B142] Zhang S. Yin Y. Jones M. B. Zhang Z. Kaiser B. L. D. Dinsmore B. A. (2015). *Salmonella* serotype determination utilizing high-throughput genome sequencing data. *J. Clin. Microbiol.* 53 1685–1692. 10.1128/JCM.00323-15 25762776 PMC4400759

[B143] Zhao S. Tyson G. H. Chen Y. Li C. Mukherjee S. Young S. (2016). Whole-genome sequencing analysis accurately predicts antimicrobial resistance phenotypes in *Campylobacter* spp. *Appl. Environ. Microbiol.* 82 459–466. 10.1128/AEM.02873-15 26519386 PMC4711122

